# Modular synthetic cross-kingdom promoters enable coordinated expression in *Escherichia coli* and *Saccharomyces cerevisiae*

**DOI:** 10.1093/nar/gkag868

**Published:** 2026-09-08

**Authors:** So-Hee Son, Soo Young Moon, Nan-Yeong An, Ju Young Lee

**Affiliations:** Graduate School of Engineering Biology, Korea Advanced Institute of Science and Technology (KAIST), Daejeon 34141, Republic of Korea; Graduate School of Engineering Biology, Korea Advanced Institute of Science and Technology (KAIST), Daejeon 34141, Republic of Korea; Graduate School of Engineering Biology, Korea Advanced Institute of Science and Technology (KAIST), Daejeon 34141, Republic of Korea; Graduate School of Engineering Biology, Korea Advanced Institute of Science and Technology (KAIST), Daejeon 34141, Republic of Korea; Department of Biological Sciences, Korea Advanced Institute of Science and Technology (KAIST), Daejeon 34141, Republic of Korea; KI for BioInnovation, Korea Advanced Institute of Science and Technology (KAIST), Daejeon 34141, Republic of Korea

## Abstract

Synthetic biology and metabolic engineering increasingly demand predictable and interoperable gene expression across phylogenetically distant organisms, as the need for portable genetic systems and transferable metabolic pathways continues to grow. However, fundamental differences in promoter architecture and transcriptional logic across kingdoms remain a key bottleneck in developing universal expression platforms. Here, we designed a set of modular hybrid promoters that enable tunable and quantitatively consistent gene expression in both *Escherichia coli* and *Saccharomyces cerevisiae*. These promoters integrate bacterial −10/−35 motifs and Shine–Dalgarno sequences with minimal yeast TATA boxes and Kozak sequences to ensure transcriptional and translational compatibility. The promoter set supported weak, moderate, and strong expression with high relative consistency across species. Applied to the biosynthetic pathway for the valuable pigment prodeoxyviolacein, the hybrid promoters enabled coordinated production in both hosts. This work establishes a broadly compatible promoter architecture and provides a foundational toolkit for cross-kingdom, multi-host synthetic biology.

## Graphical Abstract

**Graphical Abstract:**
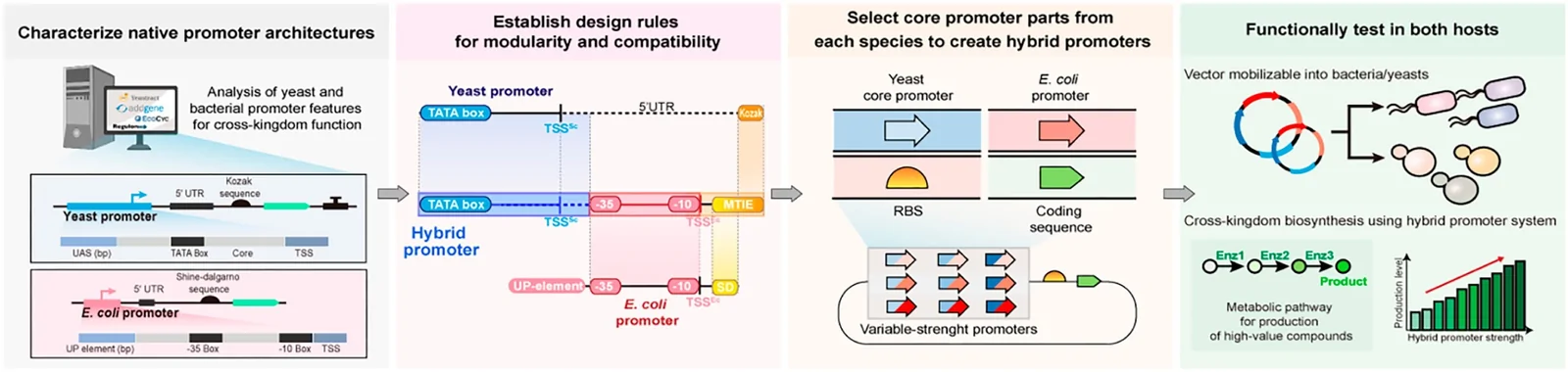

## Introduction

Genome architecture and regulatory logic for gene expression are fundamental to cellular physiology, functional diversity, and evolutionary complexity. In both prokaryotic and eukaryotic organisms, transcriptional programs are orchestrated by promoter elements that recruit RNA polymerase and associated transcription factors to initiate RNA synthesis. However, promoter sequences and regulatory mechanisms are often highly divergent across evolutionary lineages, reflecting the distinct architectures and transcriptional grammars that define different domains of life [1–3].

In nature, promoters, enhancers, and untranslated regions (UTRs) have co-evolved with distinct transcriptional machineries, resulting in functional incompatibilities between prokaryotic and eukaryotic systems [4, 5]. Consequently, gene expression systems are typically host-specific, and genetic constructs often fail to function predictably when transferred across phylogenetic boundaries. These limitations present a fundamental challenge to synthetic biology, which increasingly aims to develop portable, modular genetic systems that generalize across phylogenetically diverse organisms. Furthermore, an inability to reliably predict the quantitative behavior of genetic elements across different species continues to constrain the rational design of portable biological systems.

To address this limitation, recent efforts in synthetic biology have focused on identifying and engineering natural or synthetic DNA elements to develop broadly compatible promoter architectures, often by incorporating regulatory elements and ribosome-binding sequences from diverse sources [6–10]. These synthetic promoters aim to enable consistent, portable gene expression across phylogenetically distant species by exploiting conserved functional elements and minimizing host-specific dependencies. Substantial progress has been made in developing cross-species expression systems. For example, a T7 promoter-based bacterial expression resource enabled genetic circuits and metabolic pathways to function across multiple bacterial hosts [9]. More recently, synthetic 5′-UTRs that combine prokaryotic and eukaryotic regulatory features have demonstrated transcriptional functionality in multiple organisms [8]. However, their expression strengths often vary significantly between species, limiting their consistency and standardization. Still, the design principles, structural constraints, and quantitative performance metrics of cross-kingdom functional promoters remain insufficiently characterized (Supplementary Table S1) [9, 11].

**Figure 1.**
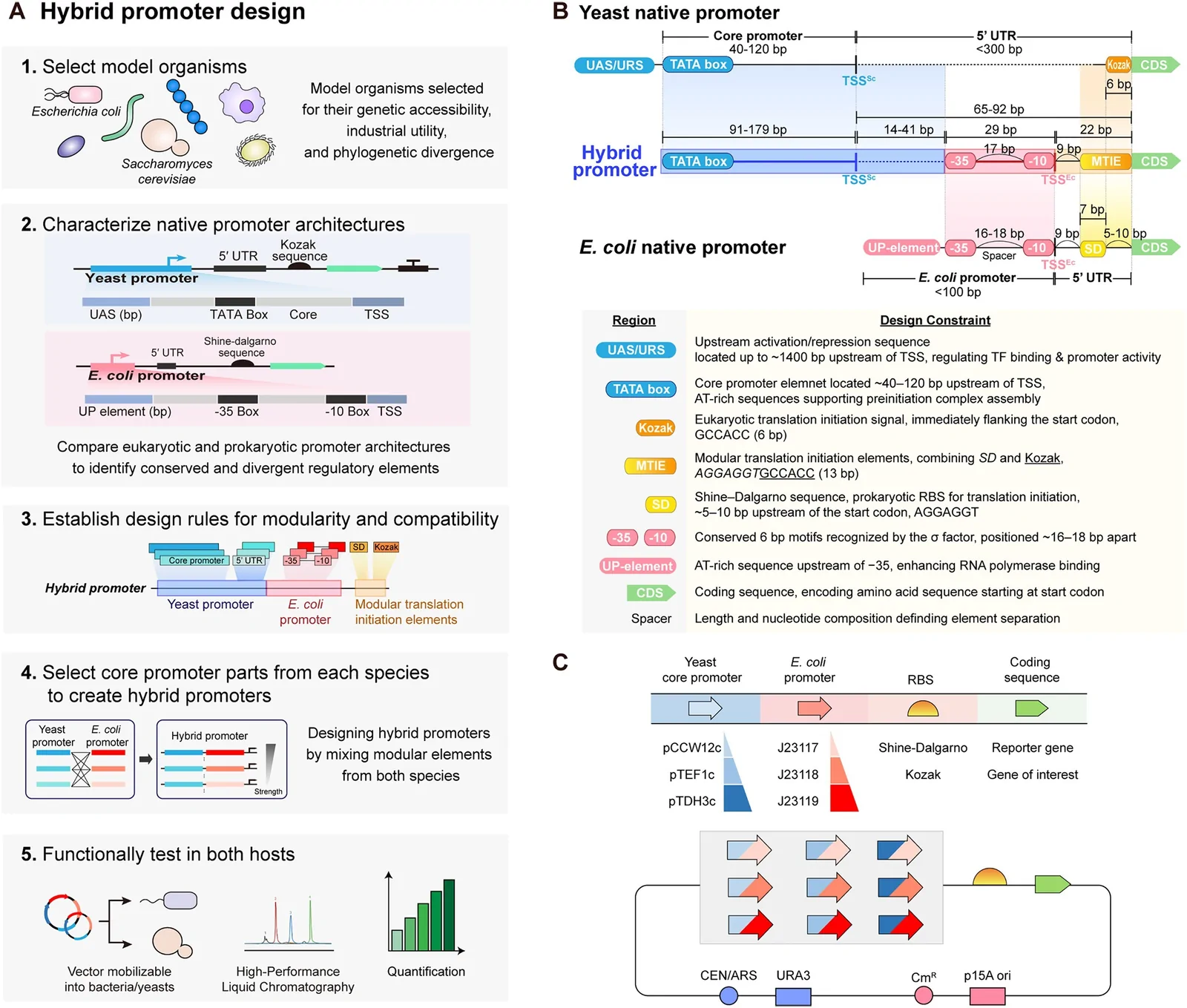
Modular architecture and design rules for cross-kingdom hybrid promoters. (**A**) Workflow for hybrid promoter design and evaluation. Promoter elements of *S. cerevisiae* and *E. coli* promoters were systematically characterized to establish modular design rules. Compact hybrid promoters were then designed and evaluated in both hosts to validate cross-kingdom compatibility and provide a quantitative framework for promoter design. (**B**) Design rules and modular assembly of hybrid promoters. Hybrid promoters were constructed by combining essential transcriptional and translational elements from both species. From *S. cerevisiae*, truncated core promoters containing canonical or non-canonical TATA boxes with preserved TATA–TSS spacing (∼40–120 bp) ensured compatibility with yeast transcription. From *E. coli*, conserved −10/−35 motifs separated by a 17-bp spacer enabled $\sigma^{70}$-dependent transcription initiation. For translation, an SD sequence located ∼5–10 bp upstream of the start codon and a Kozak consensus (GCCACC) flanking the start codon were incorporated. Bacterial promoter fragments were inserted into the yeast 5′-UTR region, thereby preserving the native 5′-UTR length and configuration and allowing modular integration of bacterial elements without disrupting yeast transcription. (**C**) Construction of the hybrid promoter series by modular assembly. Nine synthetic hybrid promoters were generated by systematically combining truncated yeast core promoters (pTDH3_C_, pTEF1_C_, and pCCW12_C_) with bacterial promoters (J23117, J23118, and J23119). This modular assembly generated compact, versatile promoter units compatible with the transcriptional machinery of both *S. cerevisiae* and *E. coli*, enabling cross-kingdom expression and providing a tunable hybrid promoter system.

To address this challenge, we developed a modular hybrid promoter system designed to function across phylogenetically distinct hosts, specifically *Escherichia coli* and *Saccharomyces cerevisiae*. The system was systematically designed according to promoter architectures characteristic of both species and assembled using standard synthetic biology parts. These included core promoter motifs derived from *E. coli* and *S. cerevisiae*, along with translation initiation elements such as the Shine–Dalgarno (SD) sequence from *E. coli* and the Kozak consensus sequence from *S. cerevisiae*. We constructed a combinatorial set of hybrid promoters and systematically evaluated their transcriptional activity in both hosts, identifying promoter configurations that enabled robust, tunable, and quantitatively consistent gene expression across species (Fig. 1). We further validated the hybrid promoter system by applying it to a model biosynthetic pathway for prodeoxyviolacein (PDV), a valuable tryptophan-derived pigment, and achieved coordinated gene expression and PDV production in both bacterial and yeast cells. Furthermore, the hybrid promoters were transcriptionally active in *Corynebacterium glutamicum* and *Pichia pastoris*, extending their applicability beyond the model hosts used in this study and supporting the broader transferability of the proposed design framework. Together, this work defines a general design framework for cross-kingdom promoter engineering and provides a transferable promoter architecture for multi-host synthetic biology.

## Materials and methods

### Strains and media

Plasmid construction was performed in *E. coli* DH5α, and validation of the hybrid promoter system was performed in *E. coli* MG1655 (DE3). *E. coli* cells were cultivated in LB medium (10 g/l tryptone, 5 g/l yeast extract, and 10 g/l NaCl) supplemented with chloramphenicol (25 μg/ml) at 37°C.

*C. glutamicum* cells were cultivated in brain heart infusion (BHI) medium (37 g/l BHI broth) supplemented with kanamycin (25 μg/ml) at 30°C.

*S. cerevisiae* CEN.PK2-1D was used as the yeast host for all experiments. All yeast cells were cultivated in yeast synthetic complete (YSC) medium lacking uracil (0.19% yeast synthetic dropout medium without uracil and 0.67% yeast nitrogen base without amino acids) supplemented with 2% glucose at 30°C.

For *P. pastoris*, cells were cultivated in YPD medium (1% yeast extract, 2% peptone, and 2% glucose) supplemented with nourseothricin (ClonNAT, 100 μg/ml) at 30°C.

### Plasmid construction

To construct a shuttle vector functional in both *E. coli* and *S. cerevisiae*, the p15A bacterial origin of replication and the chloramphenicol resistance gene for bacterial selection were obtained from the pACYCDuet-1 vector (Novagen, Darmstadt, Germany), and the CEN/ARS element for yeast replication and the URA3 gene for auxotrophic selection were derived from the pRS416 vector [12]. These elements were subsequently combined to generate the pBY shuttle vector used for further applications. Briefly, the pACYCDuet-1 vector was digested with XbaI, and the CEN/ARS–URA3 fragment, PCR (polymerase chain reaction)-amplified from pRS416, was inserted at the XbaI restriction site to construct the pBY shuttle vector (Supplementary Table S2).

Using the pBY vector as a backbone, hybrid promoters, terminator, and reporter gene constructs were assembled by fusing yeast core promoter fragments with synthetic bacterial promoters using HiFi DNA assembly (New England Biolabs, Ipswich, MA, USA) according to the manufacturer’s instructions. Yeast core promoter fragments spanning from the TATA box to the transcription start site (TSS), along with the downstream 5′-UTR, were PCR-amplified from *S. cerevisiae* genomic DNA to generate the *TDH3, TEF1*, and *CCW12* core promoter sequences. Bacterial promoter sequences (BBa_J23119, BBa_J23118, and BBa_J23117) were introduced through synthetic primers designed according to the iGEM Registry of Standard Biological Parts (http://parts.igem.org). To enable efficient translational initiation in both hosts, the SD sequence for bacterial translation and the Kozak sequence for yeast translation were combined. The fused SD–Kozak sequence was incorporated into the oligonucleotides used for constructing hybrid promoters and positioned immediately upstream of the start codon of the coding sequence. The component sequences used for hybrid promoters are provided in Supplementary Table S3.

For the construction of the PDV biosynthetic pathway, the *vioA, vioB*, and *vioE* genes from *Chromobacterium violaceum* [13] were inserted downstream of the hybrid promoters.

To evaluate the functionality of the hybrid promoter system in *C. glutamicum* and *P. pastoris*, shuttle vectors compatible with each host were used as expression backbones. Briefly, the *C. glutamicum* shuttle vector (kindly provided by Prof. Ki Jun Jeong, KAIST) [14] was digested with KpnI and NdeI, and the hybrid promoter fragments were inserted. Similarly, the *P. pastoris* shuttle vector (kindly provided by Prof. Nam Kyu Kang, Kyung Hee University) [15] was digested with PvuI and NdeI, followed by insertion of the hybrid promoter fragments. All constructs were assembled using HiFi DNA Assembly (New England Biolabs, Ipswich, MA, USA). All plasmids and primers used in this study are listed in Supplementary Tables S2 and S4.

### Fluorescence measurement by flow cytometry

For flow cytometry analysis, *E. coli, S. cerevisiae, C. glutamicum*, and *P. pastoris* cells were inoculated from single colonies into 10 ml of LB medium, YSC-URA medium supplemented with 2% glucose, BHI medium supplemented with kanamycin (25 μg/ml), and YPD medium supplemented with nourseothricin (ClonNAT, 100 μg/ml), respectively, in 50 ml bioreactor tubes and cultivated for 24 h with shaking at 250 rpm. Cultures were incubated at 37°C for *E. coli* and 30°C for *S. cerevisiae, C. glutamicum*, and *P. pastoris*. After cultivation, cells were harvested, washed twice with phosphate-buffered saline (PBS), and fixed with 1% paraformaldehyde in PBS for 10 min at room temperature. Subsequently, cells were resuspended in PBS and diluted to an OD_600_ <0.1. mCherry fluorescence was measured using a CytoFLEX S flow cytometer (Beckman Coulter, Brea, CA, USA). Samples were excited with a 561-nm laser, and emission was collected using a 610/20 nm bandpass filter (PE channel) [16]. A minimum of 50 000 events per sample was collected and analyzed using a CytoFLEX S flow cytometer.

### Relative mRNA analysis with quantitative reverse transcription PCR (qRT-PCR)

To evaluate the gene expression levels of the reporter gene mCherry driven by hybrid promoters, *E. coli and S. cerevisiae* cells were cultured for 24 h under the same conditions as described for the flow cytometry analysis, and harvested at an OD_600_ of 4. Total RNA was extracted using the RNeasy Mini Kit (Qiagen, Hilden, Germany) following enzymatic lysis for *E. coli* cells and mechanical disruption with Lysing Matrix C (MP Biomedicals, Irvine, CA, USA) using a FastPrep-24 5G homogenizer (MP Biomedicals, Irvine, CA, USA) for *S. cerevisiae* cells. Residual genomic DNA was removed by treatment with RNase-free DNase I (Thermo Fisher Scientific, Carlsbad, CA, USA) for 1 h at 37°C. RNA quality was assessed using *A*_260_/*A*_280_ ratio (>1.8), and RNA integrity was confirmed by 2% agarose gel electrophoresis, exhibiting intact rRNA bands. RNA concentration was measured using a NanoDrop spectrophotometer (Thermo Fisher Scientific, Carlsbad, CA, USA). For each sample, 100 ng of total RNA was reverse-transcribed into complementary DNA (cDNA) using RevertAid First Strand cDNA Synthesis Kit (Thermo Fisher Scientific, Carlsbad, CA, USA) according to the manufacturer’s instructions [17].

qPCR was performed in triplicate using iTaq Universal SYBR Green Supermix (Bio-Rad Laboratories, Hercules, CA, USA) on a CFX96™ Real-Time PCR Detection System (Bio-Rad Laboratories, Hercules, CA, USA). The thermal cycling was 95°C for 30 s, followed by 40 cycles of 95°C for 5 s and 60°C for 5 s, with a melt curve analysis from 65°C to 95°C in 0.5°C increments. To ensure primer specificity, PCR products were additionally verified by melt curve analysis and electrophoresis with 2% agarose gels. For normalization, *E. coli* mRNA levels were normalized to 16S rRNA gene, and *S. cerevisiae* mRNA levels were normalized to the housekeeping gene *TAF10*. The relative transcript levels were quantified using the 2^−ΔΔCT^ method. Gene-specific primers are listed in Supplementary Table S5.

### β-Galactosidase activity assay

β-Galactosidase activity was quantified using a β-Galactosidase Reporter Assay Kit (APExBIO, Houston, TX, USA) according to the manufacturer’s instructions with minor modifications [18, 19]. The hybrid promoter was placed upstream of the *lacZ* reporter gene, and the assay was based on the hydrolysis of o-nitrophenyl-β-d-galactopyranoside (ONPG) by β-galactosidase, producing the yellow product o-nitrophenol, which was quantified by measuring absorbance at 420 nm.

Specifically, *E. coli* and *S. cerevisiae* cells were cultured for 24 h under the same conditions as those used for flow cytometry analysis. For *E. coli*, cells equivalent to 1 OD_600_ were harvested by centrifugation and resuspended in 100 μl of the supplied lysis buffer. For *S. cerevisiae*, cells equivalent to 10 OD_600_ were harvested and resuspended in 0.5 ml of the supplied lysis buffer containing Lysing Matrix C (MP Biomedicals, Irvine, CA, USA). Yeast cells were mechanically disrupted using a FastPrep-24 5G homogenizer (MP Biomedicals, Irvine, CA, USA) according to the manufacturer’s protocol. Cell lysates were clarified by centrifugation, and the resulting supernatants were used to measure β-galactosidase activity. Briefly, 50 μl of the supernatant was mixed with 50 μl of Assay Reagent in a 96-well plate and incubated at 37°C for 30 min. The reaction was terminated by adding 150 μl of Stop Buffer, and absorbance was measured at 420 nm using a microplate reader.

### Flask fermentation, extraction, and quantification of PDV

For PDV production, *E. coli* cells were inoculated into 250 ml baffled flasks containing 50 ml of LB medium and incubated at 37°C and 250 rpm for 24 h. *S. cerevisiae* cells were inoculated into 250 ml baffled flasks containing 50 ml of YSC-URA medium supplemented with 608 mg/l tryptophan and 40 μM ammonium iron sulfate [20] and then incubated at 30°C with shaking at 250 rpm for 144 h.

PDV production was quantified as described previously [13]. Briefly, *E. coli* cells at an OD_600_ of 4 and *S. cerevisiae* cells at an OD_600_ of 40 were harvested by centrifugation at 13 000 rpm for 5 min. The cell pellets were resuspended in 0.5 ml of methanol containing Lysing Matrix C (MP Biomedicals, Irvine, CA, USA) and disrupted using a FastPrep-24 5G homogenizer (MP Biomedicals, Irvine, CA, USA) according to the manufacturer’s protocol [21–23]. Cell debris was removed by centrifugation, and the obtained supernatant containing PDV was analyzed using high-performance liquid chromatography (HPLC) equipped with a UV detector monitored at 610 nm. Separation was performed on a Zorbax Eclipse XDB-C18 column (4.6 × 150 mm; Agilent Technologies Inc., Santa Clara, CA, USA) using solvent A (water with 0.1% acetic acid) and solvent B (methanol with 0.1% acetic acid) at a flow rate of 1.0 ml/min. The gradient was programmed as follows: 0–5 min, linear gradient from 100% to 20% solvent A; 5–8 min, isocratic at 20% solvent A; 8–12 min, linear gradient from 20% to 100% solvent A; and 12–15 min, isocratic at 100% solvent A. All experiments were performed in three independent biological replicates.

## Results

### Design rules for cross-kingdom promoter architecture

To design a modular hybrid promoter system capable of functioning across biological kingdoms, we first established design rules for promoter architecture underlying genetic layout configurations. These included the sequence composition and length of the key functional elements, their spatial organization, and inter-element spacing, which are all features known to influence promoter function across divergent transcriptional systems (Fig. 1A and B) [8, 24]. In this study, *S. cerevisiae* and *E. coli* were selected as the model hosts due to their broad metabolic capabilities, genetic amenability to manipulation, and widespread use as chassis organisms in synthetic biology and industrial biotechnology [25–29]. Moreover, the considerable evolutionary distance between *S. cerevisiae* and *E. coli* provides an ideal framework to rigorously assess the functional portability and robustness of our modular promoter designs across phylogenetically distant systems [8].

First, we examined the promoter architectures of *S. cerevisiae* and *E. coli* to define shared and divergent features relevant to cross-kingdom promoter function. The *S. cerevisiae* promoters consist of multiple essential elements required for precise gene expression, including a core promoter region, upstream activation and repression sequences (UAS and URS, respectively), and nucleosome-disfavoring sequences such as poly(dA:dT) tracts that facilitate transcription by reducing nucleosome occupancy (Fig. 1B) [30, 31]. The core promoter region typically includes a TATA box located ∼40–120 nucleotides upstream of the TSS, often flanked by AT-rich sequences that support preinitiation complex assembly. UAS and URS elements, generally located up to approximately −1400 relative to the TSS, are positioned further upstream and modulate transcription factor binding [31–33]. While the precise length of UAS elements remains undefined, a previous study has shown that minimal synthetic UAS elements, when combined with core promoter regions, can support functional promoter activity [7]. In contrast, *E. coli* promoters exhibit a more compact and conserved architecture, usually spanning <100 bp and consisting primarily of −35 and −10 elements recognized by the σ subunit of RNA polymerase (Fig. 1B) [34, 35]. These elements are separated by an optimal spacer of 16–18 bp, forming a well-defined architecture that facilitates RNA polymerase recruitment and open complex formation [34, 36, 37]. In addition, translation initiation elements that enhance ribosome binding and scanning differ between *E. coli* and *S. cerevisiae. E. coli* utilizes the SD sequences, a purine-rich motif located ∼5–10 nucleotides upstream of the start codon, while *S. cerevisiae* relies on the Kozak consensus sequence, which flanks the start codon (Fig. 1B).

Given these foundational principles, we sought to rationally design hybrid promoters by selectively combining essential structural elements from both *S. cerevisiae* and *E. coli* promoter architectures to satisfy the structural and functional requirements of both species (Fig. 1B). For hybrid promoter design, we selected three representative constitutive promoters from each species, chosen for their robust expression and frequent use in synthetic biology and metabolic engineering. Specifically, we utilized the *S. cerevisiae TDH3, TEF1*, and *CCW12* promoters, and the *E. coli J23117, J23118*, and *J23119* promoters (Fig. 1C). Additionally, to support translation in both species, we selected two widely used modular translation initiation elements: the SD sequence (AGGAGGT) for ribosome binding in *E. coli* [38–40] and the Kozak consensus sequence (GCCACC) to enhance start codon recognition in *S. cerevisiae* (Fig. 1C) [41].

### Modular assembly of bacterial and yeast core elements for hybrid promoters

We next designed a set of synthetic hybrid promoters by systematically combining the essential core elements from *S. cerevisiae and E. coli*, aiming to generate compact and modular units that support gene expression in both species. This approach was designed to enable combinatorial flexibility while maintaining functional compatibility with the transcriptional machinery of each species. Previous studies have shown that A/T-rich motifs within yeast promoter regions can weakly mimic bacterial $\sigma^{70}$ recognition sites, thereby allowing partial transcriptional initiation in *E. coli* [42]. Consistent with this, we found putative *E. coli *  $\sigma^{70}$ recognition sites within the selected *S. cerevisiae TDH3, TEF1*, and *CCW12* promoter sequences, which were predicted using the *E. coli* promoter prediction tool (Supplementary Fig. S1) [43]. Thus, we began by assessing the transcriptional activity of both full-length yeast promoters and their truncated core promoters (pTDH3_C_, 118 bp; pTEF1_C_, 200 bp; and pCCW12_C_, 132 bp); the pTDH3_C_ and pCCW12_C_ preserved consensus TATA box sequences [TATA(A/T)A(A/T)(A/G)], whereas pTEF1_C_ included a non-canonical yet functionally reported TATA-like motif (5′-AATAAAAA-3′) [44, 45].

**Figure 2.**
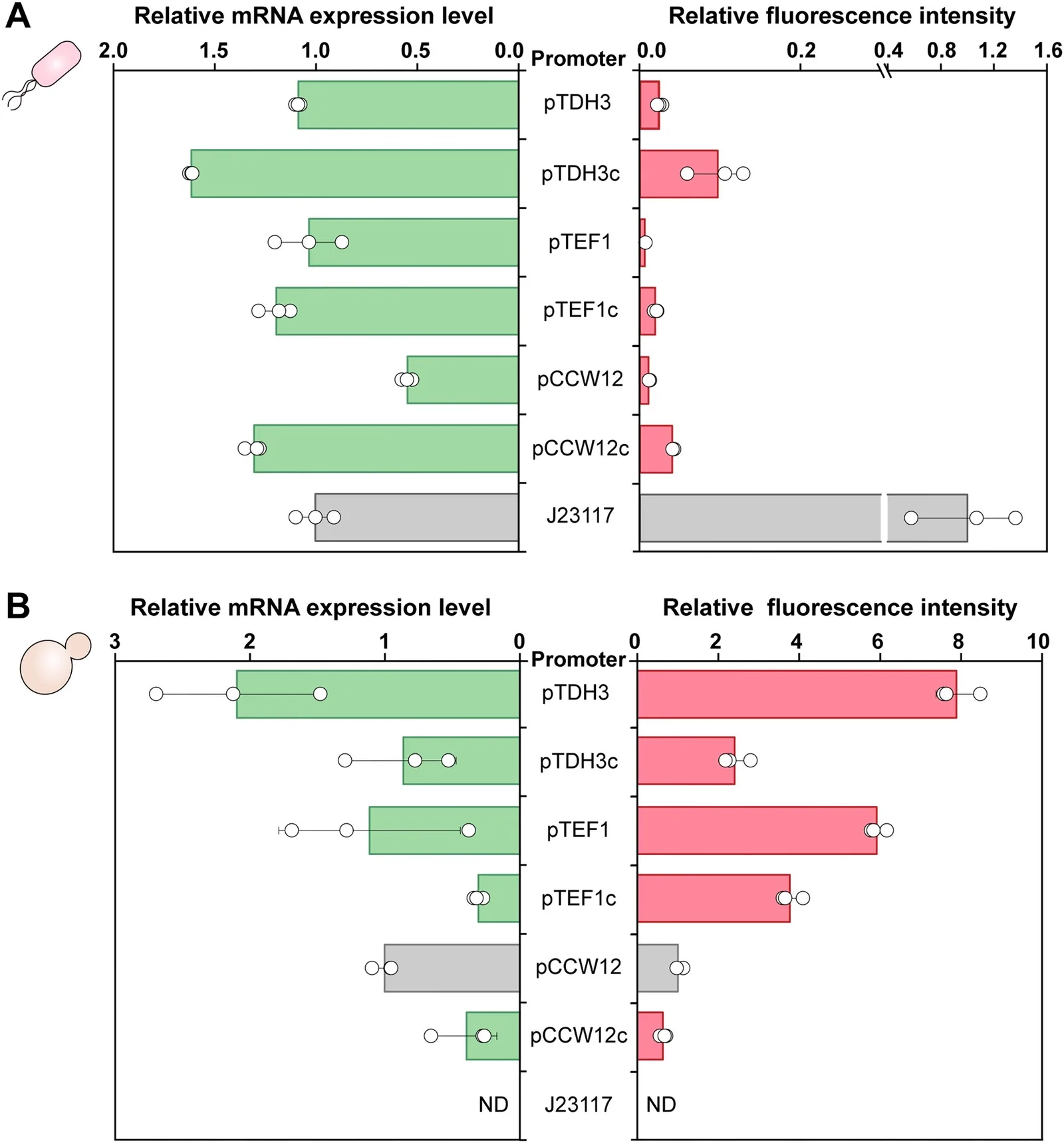
Transcriptional and translational activities of full-length and truncated yeast core promoters in *E. coli*. (**A**) Three yeast promoters (pTDH3, pTEF1, and pCCW12) and their truncated core promoters (pTDH3_C_, pTEF1_C_, and pCCW12_C_) were evaluated for mCherry expression in *E. coli*. mCherry transcript levels and fluorescence intensities were quantified, showing that yeast promoters are active in *E. coli*. Moreover, the truncated core promoters supported both transcription and translation at higher levels than their full-length counterparts. Expression levels were calculated relative to the J23117 promoter. (**B**) The same promoter set was evaluated in *S. cerevisiae*. In contrast to *E. coli*, truncated core promoters showed reduced mCherry expression at both the transcriptional and translational levels compared with their full-length promoters. Expression levels were calculated relative to the native *CCW12* promoter, and J23117-driven expression was not detected (ND). *E. coli* cells were cultured in LB medium at 37°C with shaking at 200 rpm, and *S. cerevisiae* cells were cultured in YSC-URA medium at 30°C with shaking at 250 rpm. Cells were harvested after 24 h. All data are presented as the means ± standard deviations of biological triplicates.

In line with their predicted cross-functionality, both full-length and yeast core promoters exhibited measurable transcriptional activity for the expression of the mCherry reporter gene in *E. coli* (Fig. 2A and Supplementary Table S6). Interestingly, pTDH3_C_, pTEF1_C_, and pCCW12_C_ led to 1.49-, 1.17-, and 2.38-fold increases in mCherry transcript levels, respectively, compared to their full-length counterparts. Furthermore, the corresponding mCherry fluorescence intensities increased by 4.50-, 2.00-, and 4.00-fold, respectively, indicating that the enhanced fluorescence was, to some extent, coupled with transcriptional activity. Nevertheless, the fluorescence intensities driven by these yeast core promoters in *E. coli* remained markedly lower, only ∼2%–9% of those driven by even the weak *E. coli* promoter, J23117. Additionally, we observed that the truncated yeast core promoters exhibited reduced activity in *S. cerevisiae*, compared to their full-length counterparts, as previously reported (Fig. 2B and Supplementary Table S6) [46]. Conversely, the *E. coli* J23117, J23118, and J23119 promoters from the Anderson library, which differ in $\sigma^{70}$ −10/−35 motifs and span a range of expression strengths in *E. coli*, did not exhibit detectable transcriptional activity in *S. cerevisiae*, indicating their incompatibility with the eukaryotic transcriptional machinery (Supplementary Table S6).

**Figure 3.**
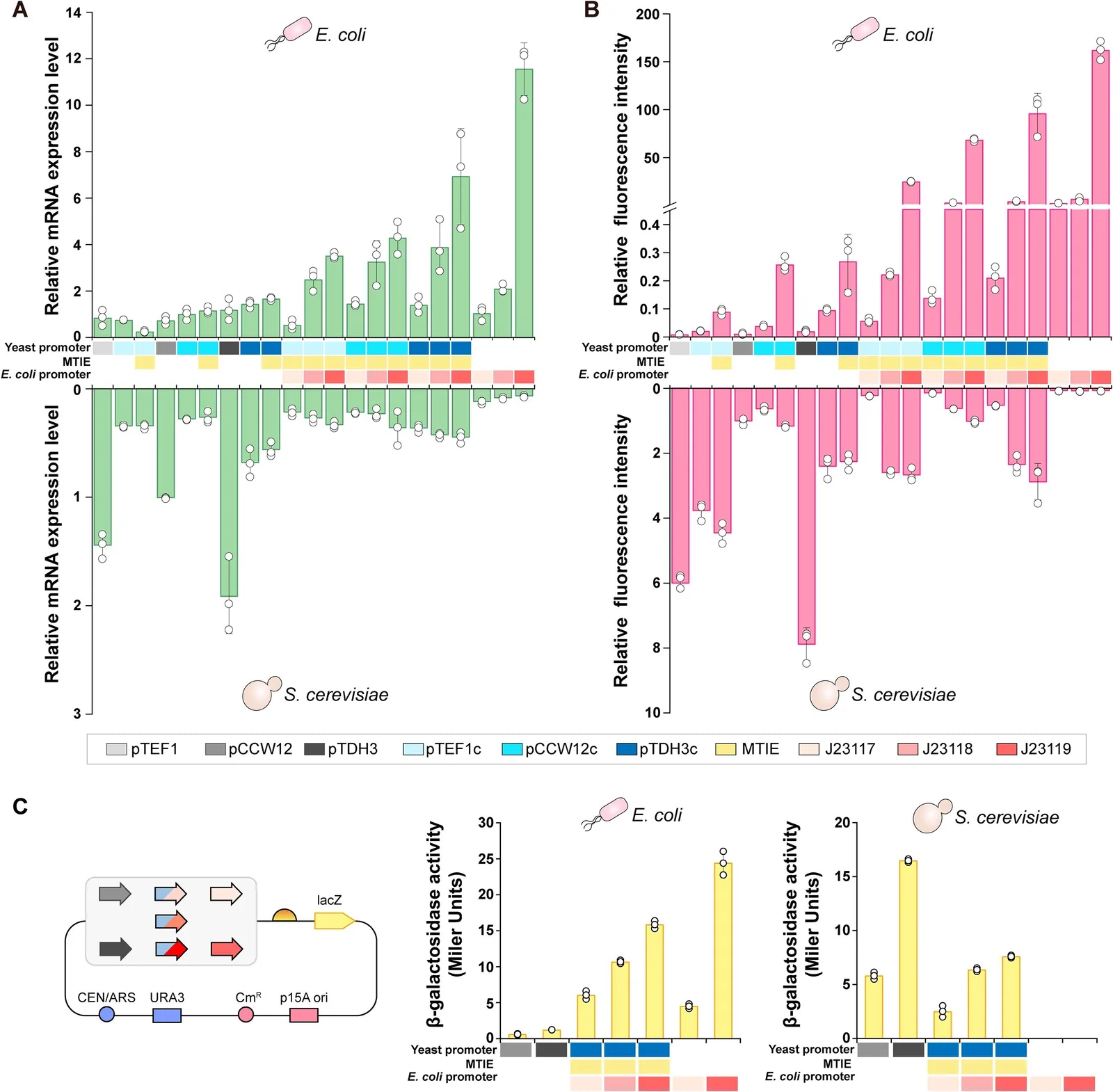
Cross-kingdom transcriptional and translational activities of hybrid promoters. A set of nine hybrid promoters, generated by combining three yeast core promoters (pTDH3_C_, pTEF1_C_, and pCCW12_C_) with three *E. coli* promoters (J23117, J23118, and J23119), was tested in *E. coli* and *S. cerevisiae* to evaluate cross-kingdom expression. (**A**) mCherry transcript levels were measured in *E. coli* and *S. cerevisiae* to assess transcriptional activity of the hybrid promoters. Expression levels were normalized to the 16S rRNA gene in *E. coli* and the housekeeping gene *TAF10* in *S. cerevisiae*. (**B**) mCherry fluorescence intensities were determined in *E. coli* and *S. cerevisiae* to evaluate protein expression driven by the hybrid promoters. These hybrid promoters enabled variable-strength gene expression across both species, exhibiting a broad dynamic range from weak to strong activity. mCherry expression levels for both transcription and translation were calculated relative to the J23117 promoter in *E. coli* and the native *CCW12* promoter in *S. cerevisiae*. (**C**) β-Galactosidase activities were measured in *E. coli* and *S. cerevisiae* to further validate the expression characteristics of the hybrid promoters. The pTDH3_C_–*E. coli* hybrid promoter series exhibited the same relative promoter strength hierarchy observed with the mCherry reporter, confirming that the characteristic expression hierarchy conferred by the hybrid promoters is reproducible across different reporter genes. Data are presented as Miller units. *E. coli* was cultured in LB medium at 37°C with shaking at 200 rpm, and *S. cerevisiae* was grown in YSC-URA medium at 30°C with shaking at 250 rpm. Cells were harvested after 24 h of culture. All the data are presented as the means ± standard deviations of biological triplicates.

To overcome the limited transcriptional compatibility of native yeast and bacterial promoters across kingdoms, we constructed a series of hybrid promoters by assembling *S. cerevisiae* core promoters (pTDH3_C_, pTEF1_C_, or pCCW12_C_) with *E. coli* promoters (J23117, J23118, or J23119) (Figs 1C and 3). By using the *S. cerevisiae* core promoters, we minimized the overall sequence length, providing design flexibility to incorporate additional structural or regulatory motifs, such as UAS or URS. In these hybrid configurations, the *E. coli* promoters were positioned directly downstream of the yeast core promoters, enabling transcription initiation by *E. coli* RNA polymerase while preserving functional compatibility with the yeast transcriptional machinery (Fig. 1B). Importantly, in *S. cerevisiae*, these inserted *E. coli* promoter regions located −57 to −22 nucleotides upstream of the start codon would not be expected to function as active transcription initiation sites for the native yeast transcriptional machinery. Instead, they are transcribed as part of the 5′-UTRs, which can accommodate heterologous sequences, although their sequence composition and secondary structure may influence translational efficiency, thereby enabling the modular integration of the *E. coli* promoter elements while minimizing their impact on yeast transcription (Fig. 1B).

However, the sequence composition and secondary structure of the 5′-UTR can influence ribosome accessibility to mRNA and thereby modulate translation efficiency [47]. Indeed, a previous study predicted that J23117, J23118, and J23119 exhibit distinct RNA folding stabilities (−17.3, −10.61, and −7.58 kcal/mol, respectively) [47]. Thus, when inserted at yeast 5′-UTR-compatible positions in our hybrid promoters, these differences in intrinsic folding stability are likely to differentially affect the translational efficiency.

Finally, to ensure efficient translation in both species, we incorporated canonical translation initiation elements: an SD sequence (AGGAGGT) ∼6–8 bp upstream of the start codon for *E. coli* [48], and a Kozak consensus sequence (GCCACC) immediately flanking the start codon for *S. cerevisiae* [20, 49]. This configuration maintained optimal spacing—typically ∼5–10 nucleotides upstream of the start codon—required for prokaryotic ribosome recruitment [50, 51], while simultaneously preserving the functional integrity required for eukaryotic translation initiation. Taken together, we assembled nine synthetic hybrid promoters by combinatorially pairing three *S. cerevisiae* core promoters with three *E. coli* promoters, each incorporating SD and Kozak elements (Fig. 1B and C, and Supplementary Table S3).

### Cross-kingdom expression with hybrid promoters

To functionally evaluate the nine hybrid promoters, we expressed hybrid promoter-*mCherry* constructs using *E. coli/S. cerevisiae* shuttle vector (Fig. 3 and Supplementary Table S7). These hybrid promoters enabled variable-strength gene expression in both *E. coli* and *S. cerevisiae*, exhibiting a broad dynamic range across transcription and translation. Specifically, expression spanned 0.52- to 6.92-fold in transcription and 0.06- to 94.22-fold in translation, compared with the J23117 promoter in *E. coli*, and 0.21- to 0.45-fold in transcription and 0.14- to 2.88-fold in translation, compared with the native *CCW12* promoter in *S. cerevisiae*, respectively (Fig. 3A and B, and Supplementary Table S7). In *E. coli*, the hybrid promoters incorporating the strong native promoter, J23119, consistently exhibited higher expression than those with the weak promoter, J23117, matching the inherent relative strengths of the native J23119 and J23117 promoters.

In the pTDH3_C_–*E. coli* hybrid promoter series, mCherry transcript levels driven by pTDH3_C_–J23117, pTDH3_C_–J23118, and pTDH3_C_–J23119 increased by 1.39-, 3.87-, and 6.92-fold, respectively, relative to the native J23117 promoter. Corresponding mCherry fluorescence levels increased by 0.21-, 2.84-, and 94.22-fold. In the pTEF1_C_ –*E. coli* hybrid promoter series, mCherry transcript levels driven by pTEF1_C_–J23117, pTEF1_C_–J23118, and pTEF1_C_–J23119 increased by 0.52-, 2.48-, and 3.50-fold, and fluorescence by 0.06-, 0.22-, and 23.34-fold, respectively. Similarly, in the pCCW12_C_–*E. coli* hybrid promoter series, mCherry transcript levels increased by 1.43-, 3.24-, and 4.28-fold, and fluorescence by 0.14-, 1.47-, and 66.76-fold, respectively. All comparisons were made relative to expression driven by the native J23117 promoter (Fig. 3A and B, and Supplementary Table S7). Together, the hybrid promoter series exhibited a highly correlated expression pattern in *E. coli*, similar to that of the corresponding native *E. coli* promoters. This result indicates that expression levels across the hybrid promoter series follow the strength of the corresponding *E. coli* promoter components (J23117 < J23118 < J23119), resulting in a consistent relative promoter strength hierarchy at both the transcript and protein levels.

Meanwhile, in *S. cerevisiae*, all hybrid promoters induced transcription at comparable levels across the J23117-, J23118-, and J23119-containing variants (Fig. 3A and B, and Supplementary Table S7), whereas fluorescence increased in a stepwise manner consistent with the relative strengths of the *E. coli* promoter variants (J23117 < J23118 < J23119) (Fig. 3A and B, and Supplementary Table S7). Specifically, in the pTDH3_C_–*E. coli* hybrid promoter series, mCherry transcript levels remained relatively constant, while fluorescence increased substantially—by 0.52-, 2.35-, and 2.88-fold for the pTDH3_C_–J23117, pTDH3_C_–J23118, and pTDH3_C_–J23119 promoters, respectively—over that of the native *CCW12* promoter. A similar trend was observed for the pTEF1_C_– and pCCW12_C_–*E. coli* hybrid promoter series, where mCherry transcript levels remained comparable, yet fluorescence increased by up to 2.67-fold. All expression values were normalized to the native *CCW12* promoter.

To clarify the marked discrepancy between transcript and fluorescence levels, we investigated whether the inserted *E. coli* promoter sequences influenced either transcription initiation or translational modulation. Because the inserted *E. coli* promoter sequences are transcribed as part of the yeast 5′-UTR, we hypothesized that sequence variation within this region could affect TSS selection and/or RNA secondary structure. We therefore mapped the TSSs of three representative hybrid promoters (TDH3_C_–J23117, TDH3_C_–J23118, and TDH3_C_–J23119) using 5′ rapid amplification of cDNA ends (5′ RACE) in both *S. cerevisiae* and *E. coli* (Supplementary Fig. S2A). All three hybrid promoters exhibited identical TSSs in each host, indicating that the observed differences in fluorescence are unlikely to arise from differences in transcription initiation.

We next evaluated potential translational effects possibly mediated by the inserted *E. coli* promoter-derived sequences within the yeast 5′-UTR [52]. We predicted the RNA secondary structures of the transcripts spanning from the experimentally determined *S. cerevisiae* TSS to the nucleotide immediately upstream of the ATG start codon. The predicted minimum free energies became progressively less negative—and thus thermodynamically less stable— from TDH3_C_–J23117 (−25.00 kcal/mol) to TDH3_C_–J23118 (−19.40 kcal/mol) and TDH3_C_–J23119 (−16.70 kcal/mol) (Supplementary Fig. S2B). Notably, this reduction in secondary structure stability closely paralleled the stepwise increase in fluorescence intensity despite comparable transcript levels. In addition, the distinct, host-specific TSSs identified between *S. cerevisiae* and *E. coli* were consistent with transcription initiation originating from the corresponding promoter module in each host. These results suggest that, in *S. cerevisiae*, translational modulation, possibly mediated by 5′-UTR-like sequences derived from the *E. coli* promoter, is the predominant driver of gene expression differences observed in these hybrid promoters, rather than transcriptional changes.

To further confirm that the observed expression hierarchy was not reporter-specific, we performed a functional assay using β-galactosidase as an additional reporter gene in both hosts (Fig. 3C and Supplementary Table S8). Among the hybrid promoter series, we selected the pTDH3_C_–*E. coli* hybrid promoter series because it consistently exhibited relatively high expression levels and a clear relative expression hierarchy in both *E. coli* and *S. cerevisiae*. The pTDH3_C_–*E. coli* hybrid promoter series exhibited stepwise β-galactosidase activities consistent with the relative promoter strength hierarchy observed with the *mCherry* reporter. These results indicate that the characteristic relative expression hierarchy conferred by the hybrid promoters is reproducible across different reporter genes.

### Cross-kingdom production of high-value compounds using hybrid promoter systems

**Figure 4.**
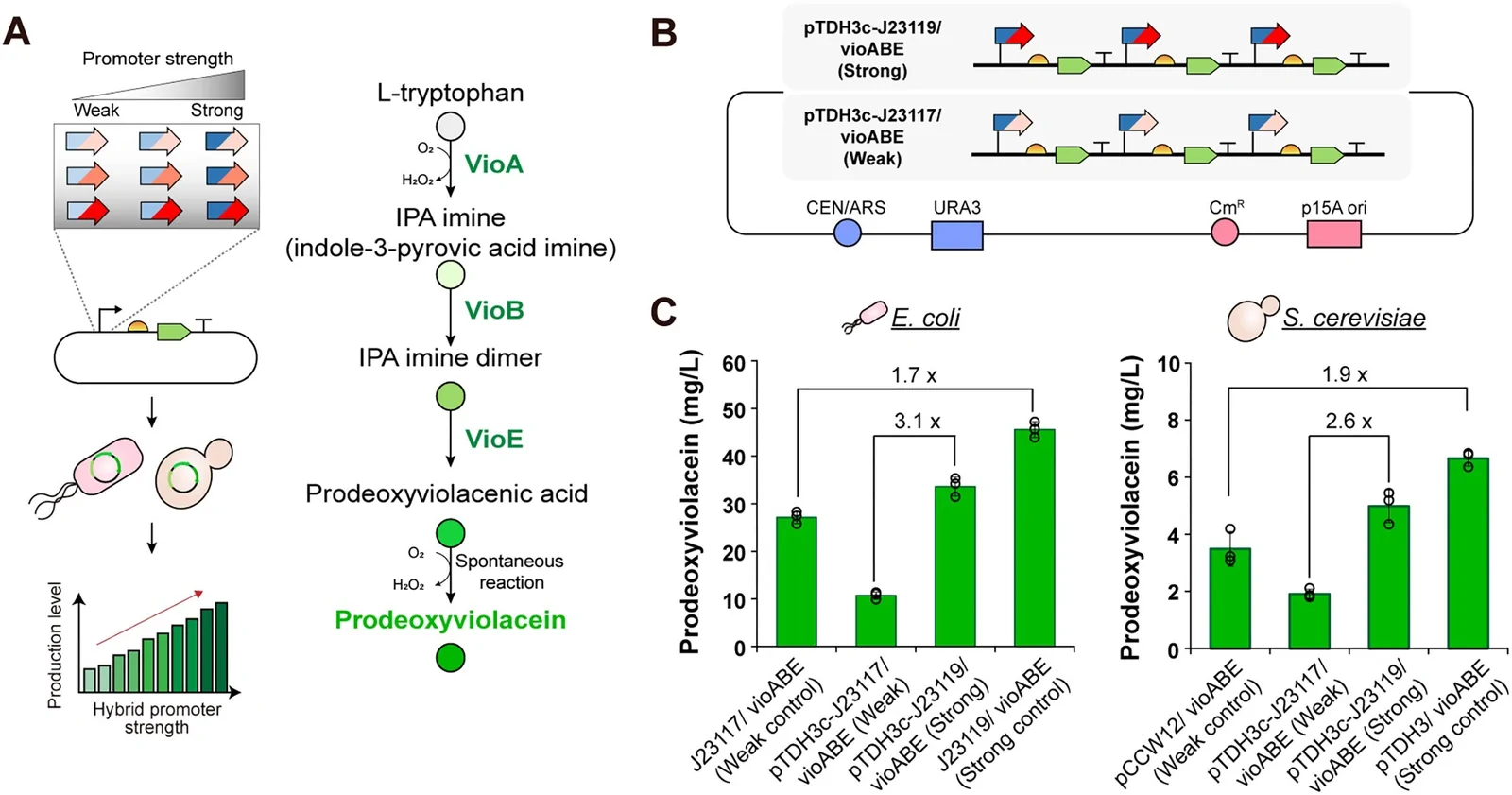
Cross-kingdom biosynthesis of PDV via hybrid promoter system. (**A**) Schematic overview of hybrid promoter–driven regulation of the PDV biosynthesis pathway. PDV was synthesized from l-tryptophan through a heterologous three-gene pathway consisting of *vioA, vioB*, and *vioE* from *C. violaceum*. (**B**) Schematic of the shuttle vector system for cross-kingdom PDV production in *E. coli* and *S. cerevisiae*. Two hybrid promoters with distinct expression strengths—a strong pTDH3_C_–J23119 and a weak pTDH3_C_–J23117—each combining bacterial and yeast core elements were employed to evaluate differential pathway expression and PDV production across the two hosts. (**C**) Quantitative analysis of PDV titers in *E. coli* and *S. cerevisiae* strains harboring hybrid promoter-driven PDV pathways. The hybrid promoters effectively coordinated PDV biosynthesis across both hosts, validating their functionality in cross-kingdom gene expression. *E. coli* was grown in LB medium at 37°C and 200 rpm for 24 h, while *S. cerevisiae* was cultured in YSC-URA medium at 30°C and 250 rpm for 144 h. All the data are presented as the means ± standard deviations of biological triplicates.

Finally, to evaluate the functional applicability of the hybrid promoter system for metabolic pathway engineering, we selected two hybrid promoters—pTDH3_C_–J23119 (strong) and pTDH3_C_–J23117 (weak)—that consistently exhibited distinct expression strengths in both *E. coli* and *S. cerevisiae* (Fig. 3). Chosen from the nine hybrid promoters, these promoters showed apparent expression differences and strong cross-species correlation, making them well-suited for assessing cross-kingdom consistency in pathway control. Then, to test these promoters, we selected the biosynthetic pathway producing PDV, a visible green pigment originally derived from *Chromobacterium violaceum* [13]. This PDV pathway converts l-tryptophan into PDV via three sequential enzymatic reactions catalyzed by VioA, VioB, and VioE (Fig. 4A).

Next, *vioA, vioB*, and *vioE* were each individually expressed under the control of either the strong pTDH3_C_–J23119 or weak pTDH3_C_–J23117 hybrid promoter using a shuttle vector. The resulting PDV-producing vectors containing pTDH3_C_–J23119/vioABE (strong) and pTDH3_C_–J23117/vioABE (weak) were introduced into both *E. coli* and *S. cerevisiae* for comparative cross-kingdom expression analysis (Fig. 4B). As expected, PDV production strongly correlated with promoter strength. The strong pTDH3_C_–J23119 promoter supported significantly higher PDV titers in both hosts—33.60 mg/l in *E. coli* and 4.99 mg/l in *S. cerevisiae* compared to the weak pTDH3_C_–J23117 promoter, which yielded 10.68 mg/l in *E. coli* and 1.91 mg/l in *S. cerevisiae* (Fig. 4C, Supplementary Fig. S3, and Supplementary Table S9). Notably, the weak-to-strong difference in PDV production observed with the hybrid promoters was comparable to, or greater than, that observed with the corresponding native promoter controls in each host. These results suggest the functional robustness and tunability of our hybrid promoter system when applied in a complex, multi-gene biosynthetic pathway across species.

Together, our results show that our hybrid promoters serve as modular and quantitative control elements that function across phylogenetically distant hosts, capable of supporting predictable expression dynamics across divergent hosts, and thereby facilitating a unified, cross-kingdom expression platform for synthetic biology.

## Discussion

Achieving compatible and well-controlled gene expression across phylogenetically distant species remains a key challenge in synthetic biology. While *E. coli* and *S. cerevisiae* are among the most widely used chassis organisms, native promoters from one species are typically non-functional or poorly functional in the other. For instance, bacterial promoters, such as those from the Anderson promoter library, are transcriptionally silent in yeast, whereas some yeast promoters can be weakly or partially active in *E. coli* (Fig. 2A) [42]. Furthermore, yeast promoters often contain extended upstream regulatory sequences, such as UAS and URS, which limit modularity and portability across hosts. These limitations impede precise control of gene expression in cross-species applications, thereby complicating the design of modular genetic constructs and constraining the transferability of metabolic pathways between organisms.

Recent studies have explored the development of cross-species or hybrid promoters capable of functioning across diverse hosts, including both prokaryotic and eukaryotic systems (Supplementary Table S1). These approaches have demonstrated that transcriptional activity can be achieved across species by combining regulatory elements from different organisms [8, 11, 42]. However, in most cases, promoter activity remains host-dependent, and it is unclear whether cross-species or hybrid promoters maintain a consistent relative strength hierarchy across different hosts. In addition, most previously reported systems have focused on reporter-level validation, without assessing whether promoter strength is preserved in a quantitative or functionally relevant manner across hosts [8, 11]. This limitation is particularly critical for applications requiring coordinated gene expression, such as multi-gene pathway engineering across species.

As a proof-of-concept study, we addressed these challenges by constructing a modular hybrid promoter system that enables transcriptional and translational compatibility in both *E. coli* and *S. cerevisiae* by combining bacterial and yeast core promoter elements. Our approach is based on identifying and integrating consensus architectural features across distinct transcriptional systems, including core promoter motifs, spacing constraints, and host-compatible translation initiation elements. As a result, these hybrid promoters preserve the essential functional features required for transcription and translation in both hosts, while maintaining a consistent relative promoter strength hierarchy (weak to strong) across both hosts, thereby enabling coordinated gene expression across phylogenetically distant organisms.

To enhance modularity, we employed minimal, truncated yeast core promoters that preserved key regulatory motifs, including canonical and non-canonical TATA boxes, while minimizing non-essential sequence length. This compact architecture enabled the successful insertion of bacterial promoter elements and allowed future extensibility with regulatory features like UAS or URS. Strategically placing bacterial promoter elements downstream of the yeast core promoter preserved bacterial transcriptional activity while serving as 5′-UTR regions in yeast, taking advantage of the compatibility of yeast with heterologous UTR sequences without disrupting transcriptional functionality. To further ensure translation in both hosts, we incorporated SD and Kozak sequences at their respective canonical positions, preserving ribosomal recruitment requirements, maintaining optimal spacing for ribosome recruitment in both prokaryotic and eukaryotic systems.

The TSS analyses further revealed that, in both *S. cerevisiae* and *E. coli*, transcription consistently initiated from the corresponding promoter module, suggesting that each host selectively recognizes its cognate promoter module within the hybrid promoter architecture. Consequently, the inserted *E. coli* promoter sequence functions as an active transcriptional promoter in *E. coli*, whereas in *S. cerevisiae*, it is transcribed as part of the yeast 5′-UTR. These findings provide mechanistic support for our modular hybrid promoter design and offer a general design principle for engineering promoter architectures that function across phylogenetically distant organisms.

Importantly, our study extends beyond reporter-based validation by demonstrating functional relevance at the metabolic pathway level. To validate the versatility of our hybrid promoter system, we evaluated its functionality across two hosts and applied our system to a three-gene biosynthetic pathway for PDV production. Using the strong (pTDH3_C_–J23119/vioABE) and weak (pTDH3_C_–J23117/vioABE) hybrid constructs, we achieved differential control of pathway flux in both *E. coli* and *S. cerevisiae*, yielding consistent, promoter-strength-dependent PDV titers. These results suggest that our hybrid promoter system can enable the simultaneous implementation and evaluation of multi-gene pathways across species, highlighting its potential as a unified, modular framework for multi-host metabolic engineering.

**Figure 5.**
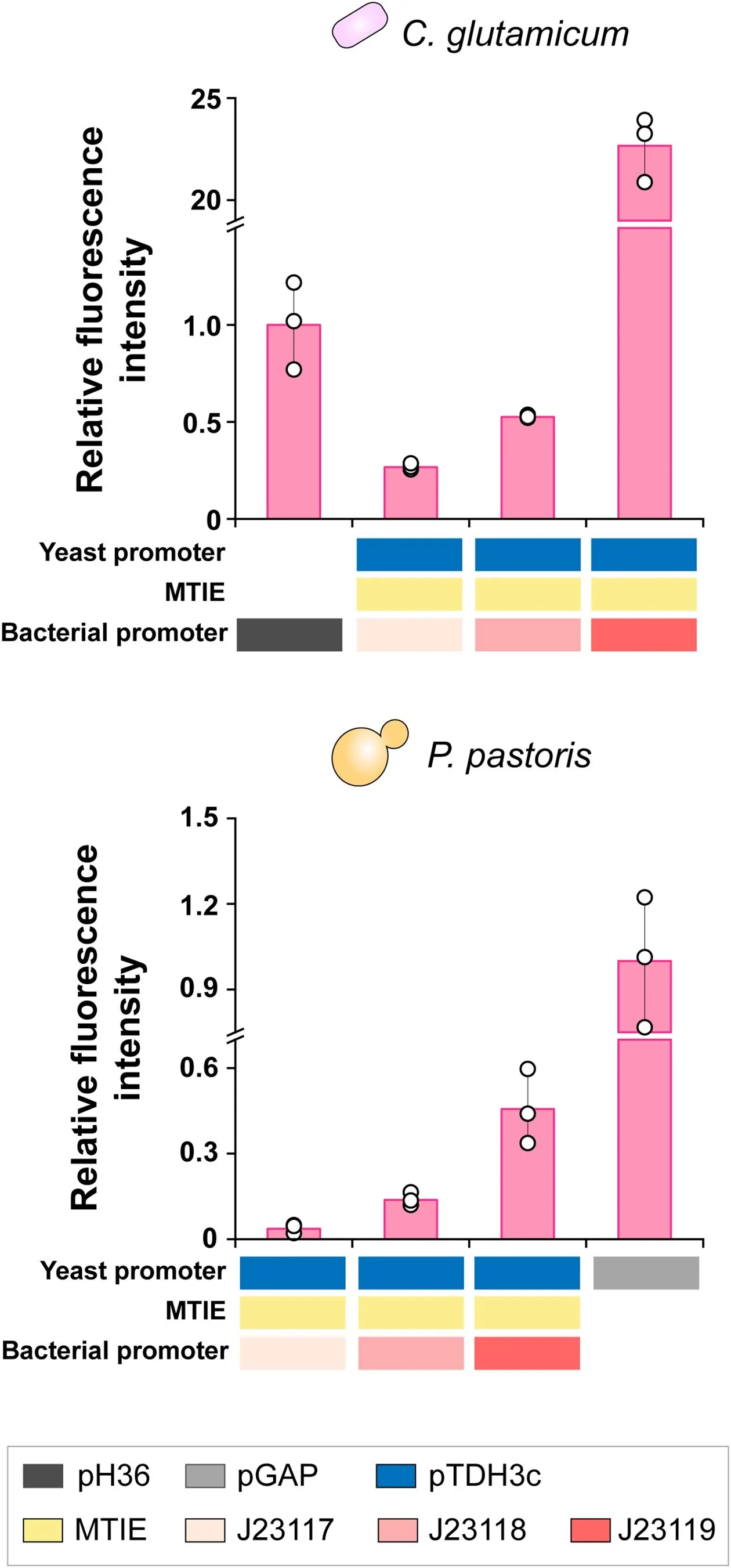
Functional validation of hybrid promoters in *C. glutamicum* and *P. pastoris*. Representative hybrid promoters were evaluated in *C. glutamicum* and *P. pastoris* to assess the applicability of the hybrid promoter system beyond the model organisms. mCherry fluorescence intensities were measured to evaluate protein expression driven by the hybrid promoters. The hybrid promoters drove gene expression in both hosts while maintaining the relative promoter strength hierarchy observed in *E. coli* and *S. cerevisiae*. mCherry fluorescence intensities were calculated relative to the native pH36 promoter in *C. glutamicum* and the native GAP promoter in *P. pastoris*.

**Figure 6.**
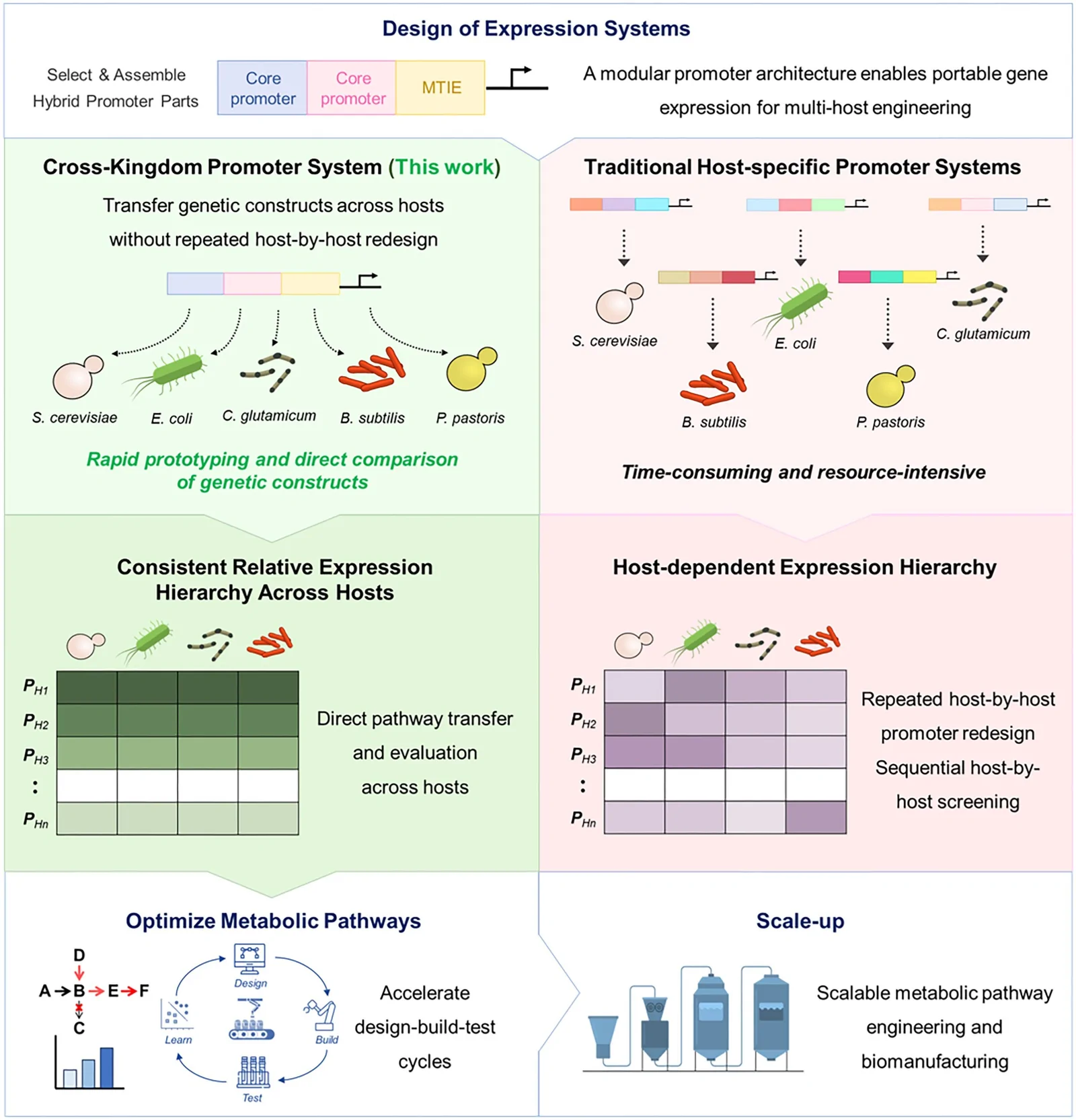
Proposed framework for multi-host pathway engineering using cross-kingdom hybrid promoters. The workflow illustrates how the modular hybrid promoter system can accelerate metabolic engineering across diverse microbial hosts. While conventional methods require repeated host-by-host redesign of promoter systems due to distinct host-specific expression characteristics, a compatible hybrid promoter architecture preserves a consistent relative expression hierarchy across diverse microbial hosts. This preserved relative hierarchy enables direct comparison of genetic constructs, rapid host selection, and efficient pathway transfer without redesigning gene expression systems for each host. Consequently, this framework minimizes iterative host-by-host redesign, supporting scalable pathway development and automated strain engineering within biofoundry pipelines.

To further assess the generality of our hybrid promoter system, we additionally evaluated representative hybrid promoters in the Gram-positive bacterium *C. glutamicum* and the methylotrophic yeast *P. pastoris* (Fig. 5 and Supplementary Table S10). Despite the evolutionary distance and distinct transcriptional machineries of these hosts, the hybrid promoters drove gene expression while preserving the relative promoter strength hierarchy observed in *E. coli* and *S. cerevisiae* (Fig. 3). While host-specific promoter optimization remains essential for achieving maximal expression in a given organism, our approach offers a complementary strategy for multi-host and cross-kingdom engineering workflows, where portability and consistency across hosts are critical. In particular, the use of a compatible promoter architecture that preserves the relative expression hierarchy across hosts can facilitate rapid prototyping and direct comparison of genetic constructs in different organisms without repeated host-by-host redesign. This capability is especially advantageous in scenarios such as multi-host pathway screening, cross-species transfer of metabolic pathways, and automated strain engineering workflows within biofoundry pipelines (Fig. 6). By streamlining iterative design–build–test cycles through a unified regulatory framework, our system can accelerate the identification of optimal host-pathway combinations, which can then be further refined via subsequent host-specific optimization. Furthermore, recent advances in high-throughput sequencing (e.g. RNA-seq and TSS mapping) [53], together with computational motif discovery and design [36, 54], can facilitate the identification of key regulatory elements even in less-characterized organisms, providing a feasible starting point for extending our design strategy.

Beyond its practical application, our studies also provide a biological blueprint for understanding the modularity and evolutionary conservation of genetic architectures across kingdoms. The partial activity of truncated yeast promoters in *E. coli* and the species-specific effects of 5′-UTR secondary structures suggest that both conserved and divergent regulatory features can be strategically exploited for cross-kingdom promoter engineering. While relative promoter strengths were preserved across hosts, absolute expression levels still varied between hosts, likely due to inherent differences in transcriptional and translational architecture. Future efforts should therefore focus on integrating synthetic UAS elements, insulator elements, or inducible modules to expand the dynamic range and improve tunability.

To advance toward interoperable gene expression across species and kingdoms, a unifying design framework is required that enables the reuse of regulatory genetic elements across diverse hosts while maintaining consistent functional behavior [9]. In this regard, our modular hybrid promoter system provides a cross-kingdom expression platform that supports the reuse of shared regulatory architectures while preserving relative promoter strength across phylogenetically distant organisms.

Together, our results establish a modular and extensible promoter framework for cross-kingdom gene expression, enabling parallel deployment of genetic designs across phylogenetically distant hosts. With further tuning of promoter composition and incorporation of host-adaptive features, this hybrid promoter system can be well-suited and enhanced across diverse microbial hosts. Ultimately, this approach can facilitate efficient pathway redesign and support the construction of shared biosynthetic pipelines, streamlining pathway transfer between diverse organisms for applications ranging from metabolic engineering to synthetic biology.

## Conclusions

Synthetic biology and metabolic engineering increasingly demand well-controlled and interoperable gene expression across phylogenetically distant organisms, as the need for portable genetic systems and transferable metabolic pathways continues to grow in cross-kingdom applications. The modular hybrid promoter system reported here demonstrates a new design paradigm for portable, cross-kingdom gene regulation. By combining conserved bacterial and yeast promoter architectures, this platform enables coordinated expression across diverse hosts. This approach provides a foundational framework for developing universal regulatory toolkits, facilitating pathway transfer, and advancing chassis-independent synthetic biology. Future integration with automated biofoundry workflows in synthetic biology and metabolic engineering will further enhance standardization, scalability, and interoperability across diverse chassis organisms.

## Supplementary Material

gkag868_Supplemental_Files

## Data Availability

All data are available within the article and its online supplementary data.
