# Supplementary material for: Modular synthetic cross-kingdom promoters enable coordinated expression in Escherichia coli and Saccharomyces cerevisiae
Source: Nucleic Acids Res. 2026 Sep 8;54(17):gkag868. doi: 10.1093/nar/gkag868 (PMC13551026; doi:10.1093/nar/gkag868)
Supplement: Supplementary file 1 [file gkag868_supplemental_files.zip › Revised Supplementary information-JYL-clean.pdf]

Supplementary information for:

Modular Synthetic Cross-Kingdom Promoters Enable Coordinated Expression in *Escherichia coli* and *Saccharomyces cerevisiae*

So-Hee Son[a,1], Soo Young Moon[a,1], Nan-Yeong An[a,1], and Ju Young Lee[a,b,c*]

[a] Graduate School of Engineering Biology, Korea Advanced Institute of Science and Technology (KAIST), Daejeon, 34141, Republic of Korea
[b] Department of Biological Sciences, Korea Advanced Institute of Science and Technology (KAIST), Daejeon, 34141, Republic of Korea
[c] KI for the BioCentury, Korea Advanced Institute of Science and Technology (KAIST), Daejeon, 34141, Republic of Korea

[1] These authors contributed equally

[*]Corresponding author:
Prof. Ju Young Lee
Phone: +82-42-350-4418
E-mail: juyounglee@kaist.ac.kr

Table of Contents

Supplementary Figure S1. Predicted *E. coli* σ70 recognition sites in *S. cerevisiae* TDH3, TEF1, and CCW12 core promoters (BPROM analysis)
Supplementary Figure S2. Transcription start site mapping and predicted RNA secondary structures of the hybrid promoters
Supplementary Figure S3. High-performance liquid chromatograms of PDV produced under the control of hybrid promoters, related Fig. 4C

Supplementary Table S1. Comparison of hybrid promoter architectures enabling cross-kingdom expression
Supplementary Table S2. List of plasmids used in this study

Supplementary Table S3. Nucleotide sequences of elements constituting the hybrid promoters (provided as a separate Excel file)
Supplementary Table S4. Primers in this study

Supplementary Table S5. Primers used for RT-qPCR and target information, related to Fig. 2-3

Supplementary Table S6. Transcriptional and translational activities of full-length and truncated yeast core promoters in *E. coli* and *S. cerevisiae*, related to Fig. 2

Supplementary Table S7. Relative cross-kingdom transcriptional and translational activities of hybrid promoters in *E. coli* and *S. cerevisiae*, related to Fig. 3A and 3B

Supplementary Table S8. β-Galactosidase activities of representative promoters in *E. coli* and *S. cerevisiae*, related to Fig. 3C

Supplementary Table S9. PDV production correlates with hybrid promoter strength in *E. coli* and *S. cerevisiae*, related to Fig. 4C

Supplementary Table S10. Relative fluorescence intensities of representative promoters in *C. glutamicum* and *P. pastoris*, related to Fig. 5

Supplementary Materials and Methods 1. 5′ Rapid Amplification of cDNA Ends (5′ RACE)

Supplementary Materials and Methods 2. RNA secondary structure prediction

Supplementary Figure S1. Predicted *E. coli* σ^{70} recognition sites in *S. cerevisiae TDH3, TEF1*, and *CCW12* core promoters (BPROM analysis).

Annotations: Core promoter, TATA box, TATA-like motif, putative -35 element, putative -10 element

TDH3 promoter

TCATTATCAATACTGCCATTTCAAAGAATACGTAAATAATTAATAGTAGTGATTTTCCTAACTTTATTTAGTCAA
AAAATTAGCCTTTTAATTCTGCTGTAACCCGTACATGCCCAAAATAGGGGGCGGGTTACACAGAATATATAACAT
CGTAGGTGTCTGGGTGAACAGTTTATTCCTGGCATCCACTAAATATAATGGAGCCCGCTTTTTAAGCTGGCATCC
AGAAAAAAAAAGAATCCCAGCACCAAAATATTGTTTTCTTCACCAACCATCAGTTCATAGGTCCATTCTCTTAGC
GCAACTACAGAGAACAGGGGCACAAACAGGCAAAAAACGGGCACAACCTCAATGGAGTGATGCAACCTGCCTGGA
GTAAATGATGACACAAGGCAATTGACCCACGCATGTATCTATCTCATTTTCTTACACCTTCTATTACCTTCTGCT
CTCTCTGATTTGGAAAAAGCTGAAAAAAAAGGTTGAAACCAGTTCCCTGAAATTATTCCCCTACTTGACTAATAA
GTATATAAAGACGGTAGGTATTGATTGTAATTCTGTAAATCTATTTCTTAAACTTCTTAAATTCTACTTTTATAG
TTAGTCTTTTTTTTAGTTTTAAAACACCAAGAACTTAGTTTCGA

TEF1 promoter

ATAGCTTCAAAATGTTTCTACTCCTTTTTTACTCTTCCAGATTTTCTCGGACTCCGCGCATCGCCGTACCACTTC
AAAACACCCAAGCACAGCATACTAAATTTCCCCTCTTTCTTCCTCTAGGGTGTCGTTAATTACCCGTACTAAAGG
TTTGGAAAAGAAAAAAGAGACCGCCTCGTTTCTTTTTCTTCGTCGAAAAAGGCAATAAAAATTTTTATCACGTTT
CTTTTTCTTGAAAATTTTTTTTTTTGATTTTTTTCTCTTTCGATGACCTCCCATTGATATTTAAGTTAATAAACG
GTCTTCAATTTCTCAAGTTTCAGTTTCATTTTTCTTGTTCTATTACAACTTTTTTTACTTCTTGCTCATTAGAAA
GAAAGCATAGCAATCTAATCTAAGTTTT

CCW12 promoter

TTCGCGGCCACCTACGCCGCTATCTTTGCAACAACTATCTGCGATAACTCAGCAAATTTTGCATATTCGTGTTGC
AGTATTGCGATAATGGGAGTCTTACTTCCAACATAACGGCAGAAAGAAATGTGAGAAAATTTTGCATCCTTTGCC
TCCGTTCAAGTATATAAAGTCGGCATGCTTGATAATCTTTCTTTCCATCCTACATTGTTCTAATTATTCTTATTC
TCCTTTATTCTTTCCTAACATACCAAGAAATTAATCTTCTGTCATTCGCTTAAACACTATATCAATA

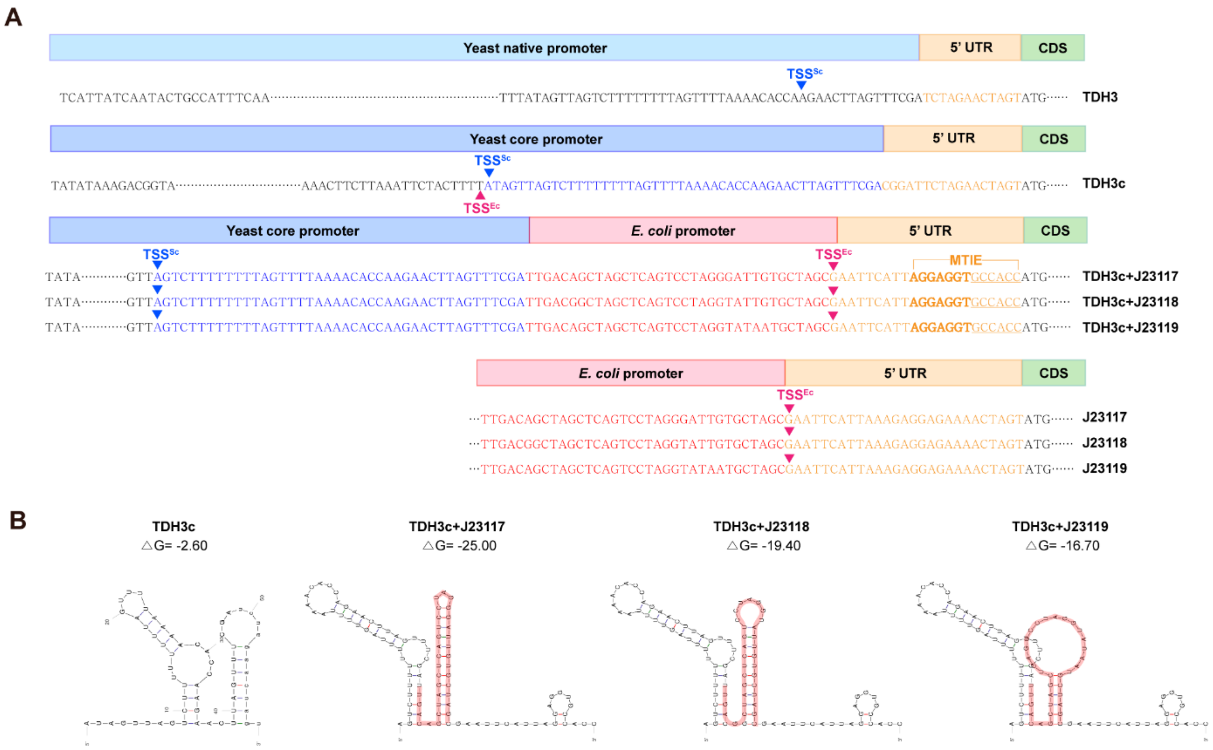

Supplementary Figure S2. Transcription start site mapping and predicted RNA secondary structures of the hybrid promoters. **(A)** Transcription start sites (TSSs) of the native TDH3 promoter, TDH3 core promoter (TDH3$_{C}$), TDH3$_{C}$–J23117, TDH3$_{C}$ –J23118, and TDH3$_{C}$ –J23119 hybrid promoters, together with the native J23117, J23118, and J23119 promoters, were mapped by 5′ RACE in *S. cerevisiae* and *E. coli*. Blue arrowheads indicate TSSs identified in *S. cerevisiae* (TSS^{Sc}), and pink arrowheads indicate TSSs identified in *E. coli* (TSS^{Ec}). The modular translation initiation element (MTIE), incorporated into the hybrid promoters, consists of the Shine–Dalgarno (SD) sequence shown in bold and the Kozak consensus sequence underlined. The native TDH3, TDH3$_{C}$, and J23117–J23119 promoters were included as controls. No reliable 5′ RACE product was obtained for the native TDH3 promoter in *E. coli*, precluding determination of its TSS in this host. **(B)** Predicted RNA secondary structures of the yeast 5′-UTRs transcribed from the TDH3$_{C}$, TDH3$_{C}$–J23117, TDH3$_{C}$–J23118, and TDH3$_{C}$–J23119 promoters. RNA secondary structures were predicted using RNA Folding Form V2.3

for transcripts spanning from the experimentally determined *S. cerevisiae* TSS to the nucleotide immediately upstream of the ATG start codon. The corresponding minimum free energies (ΔG) are shown above each predicted structure. Sequence regions corresponding to the inserted *E. coli* promoters (J23117, J23118, and J23119) are highlighted in red.

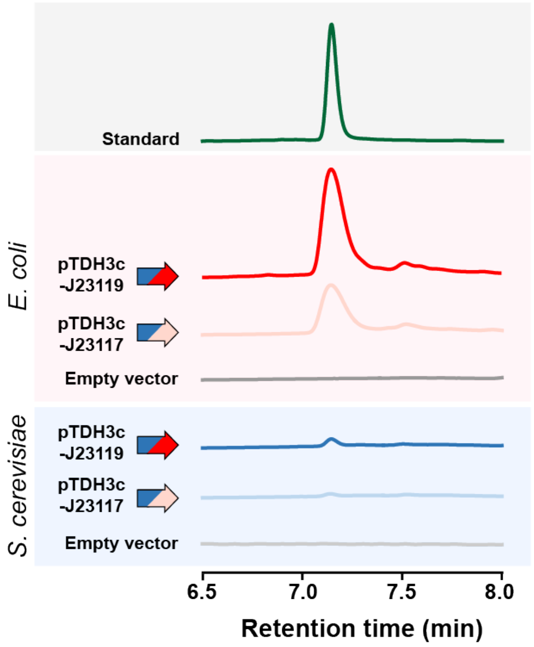

Supplementary Figure S3. High-performance liquid chromatograms of PDV produced under the control of hybrid promoters, related Fig. 4C. PDV extracted from *E. coli* expressing the strong pTDH3$_C$–J23119 (red) or weak pTDH3$_C$–J23117 (coral) hybrid promoters, and from *S. cerevisiae* expressing the strong pTDH3$_C$–J23119 (blue) or weak pTDH3$_C$–J23117 (sky blue) promoters, showed peaks corresponding to the PDV standard (green) at the same retention time. No PDV peaks were detected in the empty-vector controls (gray).

Supplementary Table S1. Comparison of hybrid promoter architectures enabling cross-kingdom expression

Expression host	Cross-kingdom expression	Transcriptional modularity		Translational modularity		Number of engineered hybrid promoters	Promoter strength	Promoter strength consistency across hosts	Metabolic pathway validation	Promoter strength consistency across hosts in metabolic pathways	Ref.
		Eukaryotic	Prokaryotic	Eukaryotic	Prokaryotic						
E. coli, *S. cerevisiae*	Yes	Yeast native and core promoters (TDH3, TEF1, CCW12, TDH3 core, TEF1 core and CCW12 core)	Bacterial promoters (J23117, J23118 and J23119)	Kozak sequence	RBS element	9	Weak / Medium / Strong	Yes	Prodeoxyviolacein	Yes	**This study**
E. coli, *B. subtilis*, *C. glutamicum*, *S. cerevisiae*, *P. pastoris*	Yes	Synthetic minimal yeast core promoter, Synthetic yeast UASs	Synthetic bacterial core promoter assembled from σ^{54}, σ^{70}, σ^{B} and σ^{D} factors-binding sequences, Bacterial UP elements library	Kozak sequence	RBS element	8	Variable	No	N/A	N/A	(1)
E. coli, *P. aeruginosa*	No	N/A	De novo AI-designed 5′ regulatory sequences	N/A	N/A	> 2,000	Variable	N/A	N/A	N/A	(2)
E. coli, *K. aerogenes*, *P. putida* *S. enterica* *B. subtilis*, *S. cerevisiae*	Yes	Synthetic minimal yeast core promoters, Minimal yeast UASs	Bacterial T7 promoter	Kozak sequences	Synthetic RBS elements	48	Variable	No	Tyrocitabine	No	(3)
E. coli, *S. cerevisiae*	Yes	Yeast GAL1/10 bidirectional promoter	N/A	N/A	N/A	1	N/A	N/A	Styrene / Cinnamyl alcohol	No	(4)
E. coli, *P. putida*, *B. subtilis*	No	N/A	Bacterial T7 promoter, TetO operator	N/A	cross-species RBS	1	Variable	N/A	Neurosporene	N/A	(5)

Note: *E. coli*, *Escherichia coli*; *S. cerevisiae*, *Saccharomyces cerevisiae; B. subtilis, Bacillus subtilis; C. glutamicum, Corynebacterium glutamicum; P. pastoris, Pichia pastoris; P. aeruginosa, Pseudomonas aeruginosa; K. aerogenes, Klebsiella aerogenes; S. enterica, Salmonella enterica;* TSSs, Transcription Start Sites; UAS, Upstream Activating Sequences; UP elements, Upstream Promoter elements; N/A, Not Applicable.

Supplementary Table S2. List of plasmids used in this study

Plasmids	Description / Genotype	Reference
pRS416	CEN/ARS ori, *URA3* marker Yeast expression vector	Mumberg et al. (6)
pACYCDuet-1	P15A ori, Cm^R *E. coli* expression vector	Novagen
pBY	CEN/ARS ori, *URA3* marker, P15A ori, Cm^R expression vector for *E. coli* and *S. cerevisiae*	This paper
pGADT7-yeCherry-yeGFP	pGADT7-ADH700-yeCherry-pTEF1(ashbya)-yeGFP-DHFR	Addgene#24585<br>Edwards et al. (7)
pTDH3-mCherry	pBY-P_{TDH3}-mCherry-T_{CYC1}	This paper
pTEF1-mCherry	pBY-P_{TEF1}-mCherry-T_{CYC1}	This paper
pCCW12-mCherry	pBY-P_{CCw12}-mCherry-T_{CYC1}	This paper
J23117-mCherry	pBY-P_{J23117}-mCherry -T_{CYC1}	This paper
J23118-mCherry	pBY-P_{J23118}-mCherry -T_{CYC1}	This paper
J23119-mCherry	pBY-P_{J23119}-mCherry -T_{CYC1}	This paper
pTDH3$_C$-mCherry	pBY-P_{TDH3c}-mCherry-T_{CYC1}	This paper
pTEF1$_C$-mCherry	pBY-P_{TEF1c}-mCherry-T_{CYC1}	This paper
pCCW12 $_C$-mCherry	pBY-P_{CCw12c}-mCherry-T_{CYC1}	This paper
pTDH3$_C$-MTIE-mCherry	pBY-P_{TDH3c}-MTIE-mCherry-T_{CYC1}	This paper
pTEF1$_C$-MTIE-mCherry	pBY-P_{TEF1c}-MTIE-mCherry-T_{CYC1}	This paper
pCCW12$_C$-MTIE-mCherry	pBY-P_{CCw12c}-MTIE-mCherry-T_{CYC1}	This paper
pTDH3$_C$-J23117-mCherry	pBY-$P_{TDH3c\text{-}J23117}$-MTIE-mCherry-T_{CYC1}	This paper
pTDH3$_C$-J23118-mCherry	pBY-$P_{TDH3c\text{-}J23118}$-MTIE-mCherry-T_{CYC1}	This paper
pTDH3$_C$-J23119-mCherry	pBY-$P_{TDH3c\text{-}J23119}$-MTIE-mCherry-T_{CYC1}	This paper
pTEF1$_C$-J23117-mCherry	pBY-$P_{TEF1c\text{-}J23117}$-MTIE-mCherry-T_{CYC1}	This paper
pTEF1$_C$-J23118-mCherry	pBY-$P_{TEF1c\text{-}J23118}$-MTIE-mCherry-T_{CYC1}	This paper
pTEF1$_C$-J23119-mCherry	pBY-$P_{TEF1c\text{-}J23119}$-MTIE-mCherry-T_{CYC1}	This paper
pCCW12$_C$-J23117-mCherry	pBY-$P_{CCW12c\text{-}J23117}$-MTIE-mCherry-T_{CYC1}	This paper
pCCW12$_C$-J23118-mCherry	pBY-$P_{CCW12c\text{-}J23118}$-MTIE-mCherry-T_{CYC1}	This paper
pCCW12$_C$-J23119-mCherry	pBY-$P_{CCW12c\text{-}J23119}$-MTIE-mCherry-T_{CYC1}	This paper
pTDH3$_C$-J23117-vioABE	pBY-$P_{TDH3c\text{-}J23117}$-MTIE-vioA-T_{ENO1}-$P_{TDH3c\text{-}J23117}$-MTIE-vioB-T_{PGK1}- $P_{TDH3c\text{-}J23117}$-MTIE-vioE-T_{ADH1}	This paper
pTDH3$_C$-J23119-vioABE	pBY-$P_{TDH3c\text{-}J23119}$-MTIE-vioA-T_{ENO1}-$P_{TDH3c\text{-}J23119}$-MTIE-vioB-T_{PGK1}-$P_{TDH3c\text{-}J23119}$-MTIE-vioE-T_{ADH1}	This paper
pTDH3-lacZ	pBY-P_{TDH3}-lacZ-T_{CYC1}	This paper
pCCW12-lacZ	pBY-P_{CCW12}-lacZ-T_{CYC1}	This paper

J23117-lacZ	pBY-P_{J23117}-lacZ-T_{CYC1}	This paper
J23119-lacZ	pBY-P_{J23119}-lacZ-T_{CYC1}	This paper
pTDH3$_C$-J23117-lacZ	pBY-$P_{TDH3c-J23117}$-MTIE-lacZ-T_{CYC1}	This paper
pTDH3$_C$-J23118-lacZ	pBY-$P_{TDH3c-J23118}$-MTIE-lacZ-T_{CYC1}	This paper
pTDH3$_C$-J23119-lacZ	pBY-$P_{TDH3c-J23119}$-MTIE-lacZ-T_{CYC1}	This paper
pHCP	pCES_PLPV derivative, pHCMS; parB nonsense mutation, Km^R for *C. glutamicum*	Choi et al. (8)
pHCP-H36-mCherry	pHCP-P_{H36}-mCherry-T_{rrnB}	This paper
pHCP-J23117-mCherry	pHCP-$P_{TDH3c-J23117}$-MTIE-mCherry-T_{rrnB}	This paper
pHCP-J23118-mCherry	pHCP-$P_{TDH3c-J23118}$-MTIE-mCherry-T_{rrnB}	This paper
pHCP-J23119-mCherry	pHCP-$P_{TDH3c-J23119}$-MTIE-mCherry-T_{rrnB}	This paper
pCast	pBR322 ori, Amp^R marker and CloNAT marker expression vector for *E. coli* and *P. pastoris*	Choi et al. (9)
pCast-GAP-mCherry	pCast-P_{GAP}-mCherry- $T_{RPS25At}$	This paper
pCast-J23117-mCherry	pCast-$P_{TDH3c-J23117}$-MTIE-mCherry-$T_{RPS25At}$	This paper
pCast-J23118-mCherry	pCast-$P_{TDH3c-J23118}$-MTIE-mCherry- $T_{RPS25At}$	This paper
pCast-J23119-mCherry	pCast-$P_{TDH3c-J23119}$-MTIE-mCherry- $T_{RPS25At}$	This paper

Supplementary Table S4. Primers in this study.

Name	Sequence (5'-3')
pBY_URA-F	ATTAATCAGATAAAATATTTCTAGATTAGTTTTGCTGGCCGCATCTTC
pBY_CEN-R	GAGATAAATTGCACTGAAATCTAGACGAAAAGTGCCACCTGGGTC
pBY_Vec-F	GGAATTGTGAGCGGATAACAATTCC
pBY_Vec-R	CATAAGGGAGAGCGTCGAGATC
J23117_mCherry-F	TCTCGACGCTCTCCCTTATGTTGACAGCTAGCTCAGTCCTAGGGATTGT GCTAGCGAATTCATTAAAGAGGAGAAAACTAGTATGGTA
J23118_mCherry-F	TCTCGACGCTCTCCCTTATGTTGACGGCTAGCTCAGTCCTAGGTATTGT GCTAGCGAATTCATTAAAGAGGAGAAAACTAGTATGGTAA
J23119_mCherry-F	TCTCGACGCTCTCCCTTATGTTGACAGCTAGCTCAGTCCTAGGTATAAT GCTAGCGAATTCATTAAAGAGGAGAAAACTAGTATGGTAA
mCherry-R	CTCGAGTTATTTGTACAATTCGTCCATTCCACCCGTTG
CCW12-F	TCTCGACGCTCTCCCTTATGTTCGCGGCCACCTACGC
CCW12c-F	TCTCGACGCTCTCCCTTATGTATATAAAGTCGGCATGCTTGATAATCTTT CTTTCC
CCW12-R	CCATACTAGTTCTAGATATTGATATAGTGTTTAAGCGAATGACAGAAG
CCW12_mCherry-F	AATATCTAGAACTAGTATGGTAAGTAAGGGTGAAGAAGACAATATGG
MTIE_mCherry-F	GAATTCATTAGGAGGTGCCACCATGGTAAGTAAGGGTGAAGAAGACA ATATGG
mCherry_tCYC-R	GACATAACTAATTACATGACTCGAGTTATTTGTACAATTCGTCCATTCC ACCCG
tCYC-F	AATTGTACAAATAACTCGAGTCATGTAATTAGTTATGTCACGCTTAC
tCYC-R	TGTTATCCGCTCACAATTCCGCAAATTAAAGCCTTCGAGCGTC
CCW12c_MTIE-R	TGGCACCTCCTAATGAATTCTATTGATATAGTGTTTAAGCGAATGACAG AAG
CCW12c_J17_MTIE-R	CATGGTGGCACCTCCTAATGAATTCGCTAGCACAATCCCTAGGACTGA GCTAGCTGTCAATATTGATATAGTGTTTAAGCGAATGACAGAAGATT
CCW12c_J18_MTIE-R	CATGGTGGCACCTCCTAATGAATTCGCTAGCACAATACCTAGGACTGA GCTAGCCGTCAATATTGATATAGTGTTTAAGCGAATGACAGAAGATT
CCW12c_J19_MTIE-R	CATGGTGGCACCTCCTAATGAATTCGCTAGCATTATACCTAGGACTGAG CTAGCTGTCAATATTGATATAGTGTTTAAGCGAATGACAGAAGATT
TDH3-F	TCTCGACGCTCTCCCTTATGTCATTATCAATACTGCCATTTCAAAG
TDH3_mCherry-F	TCGATCTAGAACTAGTATGGTAAGTAAGGGTGAAGAAGACAATATGG
TDH3-R	CCATACTAGTTCTAGATCGAAACTAAGTTCTTGGTGTTTTAAAAC
TDH3c-F	TCTCGACGCTCTCCCTTATGTATATAAAGACGGTAGGTATTGATTGTAAT TC
TDH3c_MTIE-R	CATGGTGGCACCTCCTAATGAATTCTCGAAACTAAGTTCTTGGTGTTTT AAAAC
TDH3c_J17_MTIE-R	CATGGTGGCACCTCCTAATGAATTCGCTAGCACAATCCCTAGGACTGA GCTAGCTGTCAATCGAAACTAAGTTCTTGGTGTTTTAAAAC
TDH3c_J18_MTIE-R	CATGGTGGCACCTCCTAATGAATTCGCTAGCACAATACCTAGGACTGA GCTAGCCGTCAATCGAAACTAAGTTCTTGGTGTTTTAAAACT
TDH3c_J19_MTIE-R	CATGGTGGCACCTCCTAATGAATTCGCTAGCATTATACCTAGGACTGAG CTAGCTGTCAATCGAAACTAAGTTCTTGGTGTTTTAAAAC

TEF1-F	TCTCGACGCTCTCCCTTATGATAGCTTCAAAATGTTTCTACTCC
TEF1c-F	TCTCGACGCTCTCCCTTATGAATAAAAATTTTTATCACGTTTCTTTTTCTTG
TEF1-R	CTTACCATACTAGTTCTAGAAAACTTAGATTAGATTGCTATGCTTTC
TEF1_mCherry-F	GTTTTCTAGAACTAGTATGGTAAGTAAGGGTGAAGAAGACAATATGG
TEF1c_MTIE-R	CATGGTGGCACCTCCTAATGAATTCAAAACTTAGATTAGATTGCTATGCTTTC
TEF1c_J17_MTIE-R	CATGGTGGCACCTCCTAATGAATTCGCTAGCACAATCCCTAGGACTGAGCTAGCTGTCAAAAAACTTAGATTAGATTGCTATGCTTTC
TEF1c_J18_MTIE-R	CATGGTGGCACCTCCTAATGAATTCGCTAGCACAATACCTAGGACTGAGCTAGCCGTCAAAAAACTTAGATTAGATTGCTATGCTTTC
TEF1c_J19_MTIE-R	CATGGTGGCACCTCCTAATGAATTCGCTAGCATTATACCTAGGACTGAGCTAGCTGTCAAAAAACTTAGATTAGATTGCTATGCTTTC
pBY_promoter-F	GGATCTCGACGCTCTCCCTTATG
Promoter-MTIE-R	CATGGTGGCACCTCCTAATGAATTC
lacZ_TDH3-F	GTTTCGAAGGAGGTGCCACCATGATTACGGATTCACTGGCCGT
lacZ_CCW12-F	ATCAATAAGGAGGTGCCACCATGATTACGGATTCACTGGCCGT
lacZ-R	GTTTCTTTACCAGACTCGAGTTATTTTTGACACCAGACCAACTGGTAATGG
J23117-lacZ-F	CAGGATCCGAATTCGAGCTCTTGACAGCTAGCTCAGTCCTAGGGATTGTGCTAGCGAATTCATT
J23119-lacZ-F	CAGGATCCGAATTCGAGCTCTTGACAGCTAGCTCAGTCCTAGGTATAATGCTAGCGAATTCATT
MTIE-lacZ-F	GAATTCATTAGGAGGTGCCACCATGATTACGGATTCACTGGCCGT
TDH3c-F	CAGGATCCGAATTCGAGCTCTATATAAAGACGGTAGGTATTGA
MTIE-lacZ-R	GCCAGTGAATCCGTAATCATGGTGGCACCTCCTAATGAATTC
MTIE_VioA_tENO1-F	GAATTCATTAGGAGGTGCCACCATGAAACATTCTTCCGATATCTGCATTGTT
VioA_tENO1-R	TGTTATCCGCTCACAATTCCGGTACCGGCCATACATGGGT
TDH3c_VioB-F	AAGGAGATATACATATGGCAGATCTTATATAAAGACGGTAGGTATTGATTGTAATTCTGTAAATC
MTIE_VioB_tPGK1-F	GAATTCATTAGGAGGTGCCACCATGAGCATTCTGGATTTCCCGC
VioB_tPGK1-R	CCGATATCCAATTGAGATCTTTGGGTACCGGCCACATAGAAAT
TDH3c_VioE-F	TCGTCTACTAGCGCAGCTTAATTAATATATAAAGACGGTAGGTATTGATTGTAATTCTGTAAATC
MTIE_VioE-F	GAATTCATTAGGAGGTGCCACCATGGAGAACCGTGAGCCACC
VioE-R	GAAATTCGCGCCACTCGAGTTATTAGCGCTTGGCCGCGAAAA
VioE_tADH1-F	TAATAACTCGAGTGGCGCGAATTTCT
VioE_tADH1-R	GGTGGCAGCAGCCTAGGTTAATTAACCGGTAGAGGTGTGGTCAATAAGAG
J23117_VioA-F	GAATTCGAGCTCTTGACAGCTAGCTCAGTCCTAGGGATTGTGCTAGCGAATTCATTAGGAGGTGCCACCATGAAACATTCTTCCGATATCTGCATTGTT
J23117_VioA-R	GCTAGCACAATCCCTAGGACTGAGCTAGCTGTCAAAGATCTGCCATATGTATATCTCCTTCTTATACTTAACTAATATAC

J23117_VioB-F	AAGGAGATATACATATGGCAGATCTTTGACAGCTAGCTCAGTCCTAGG GATTGTGCTAGC
J23117_VioB-R	GCTAGCACAATCCCTAGGACTGAGCTAGCTGTCAATTAATTAAGCTGC GCTAGTAGACGAGTCCATGTGCTGGCGTTCAA
J23117_VioE-F	GCCAGCACATGGACTCGTCTACTAGCGCAGCTTAATTAATTGACAGCT AGCTCAGTCCTAGGGATTG
J23119_VioA-F	GAATTCGAGCTCTTGACAGCTAGCTCAGTCCTAGGTATAATGCTAGCG AATTCATTAGGAGGTGCCACCATGAAACATTCTTCCGATATCTG
J23119_VioA-R	GCTAGCATTATACCTAGGACTGAGCTAGCTGTCAAAGATCTGCCATATG TATATCTCCTTCTTATACTTAACTAATATAC
J23119_VioB-F	AAGGAGATATACATATGGCAGATCTTTGACAGCTAGCTCAGTCCTAGGT
J23119_VioB-R	GCTAGCATTATACCTAGGACTGAGCTAGCTGTCAATTAATTAAGCTGCG CTAGTAGACGAGTCCATGTGCTGGCGTTCAA
J23119_VioE-F	TGAACGCCAGCACATGGACTCGTCTACTAGCGCAGCTTAATTAATTGA CAGCTAGCTCAGTCCTAGGT
VioE-R	CAGCGGTTTCTTTACCAGACTCGAGCCGGTAGAGGTGTGGTCAATAAG AGCG
Hybrid_Coryne-F	GGGAACAAAAGCTGGGTACCTATATAAAGACGGTAGGTATTGATTGTA ATTCTGTAA
Hybrid_Coryne-R	TCACCCTTACTTACCATATGGGTGGCACCTCCTAATGAATTC
Hybrid_Pichia-F	GTTTTTTCGCGATCGTATATAAAGACGGTAGGTATTGATTGTAATTCTGT AAATCTA
Hybrid_Pichia-R	CCATGGTGGCACCTCCTAATGAATTC
Pichia_mCherry-F	GAATTCATTAGGAGGTGCCACCATGG
Pichia_mCherry-R	GCGTACCTTCATATGGTCTACCTTCACCTT
mCherry-5RACE-GSP1	GATTACGCCAAGCTTCGTCCATTCCACCCGTTGAGTGCCTACC
16S_rRNA_qPCR-F	AGGCCTTCGGGTTGTAAAGT
16S_rRNA_qPCR-R	CGGGGATTTCACATCTGACT
TAF10_qPCR-F	ATATTCCAGGATCAGGTCTTCCGTAGC
TAF10_qPCR-R	GTAGTCTTCTCATTCTGTTGATGTTGTTGT
mCherry_qPCR-F	ACGGCGGAGTTGTTACTGTT
mCherry_qPCR-R	CATACGTTCAGATGAGGCTTCC

Supplementary Table S5. Primers used for RT-qPCR and target information, related to Fig. 2-3.

**Name**	**Sequence (5'-3')**	**Amplicon length (bp)**	**Source**
16S_rRNA_qPCR-F	AGGCCTTCGGGTTGTAAAGT	201	NC_000913.3
16S_rRNA_qPCR-R	CGGGGATTTCACATCTGACT		
TAF10_qPCR-F	ATATTCCAGGATCAGGTCTTCCGTAGC	141	NC_001136.10
TAF10_qPCR-R	GTAGTCTTCTCATTCTGTTGATGTTGTTGT		
mCherry_qPCR-F	ACGGCGGAGTTGTTACTGTT	149	Addgene#24585 Edwards et al. (7)
mCherry_qPCR-R	CATACGTTCAGATGAGGCTTCC		

Supplementary Table S6. Transcriptional and translational activities of full-length and truncated yeast core promoters in *E. coli* and *S. cerevisiae*, related to Fig. 2.

	E. coli		***S. cerevisiae***	
	**Relative mRNA expression level**	**Relative fluorescence intensity**	**Relative mRNA expression level**	**Relative fluorescence intensity**
**pTDH3**	1.09 (±0.01)	0.02 (±0.01)	2.10 (±0.61)	7.88 (±0.51)
**pTDH3$_{C}$**	1.62 (±0.01)	0.09 (±0.03)	0.86 (±0.39)	2.40 (±0.33)
**pTEF1**	1.03 (±0.31)	0.01 (±0.00)	1.11 (±0.67)	5.91 (±0.21)
**pTEF1$_{C}$**	1.20 (±0.08)	0.02 (±0.00)	0.31 (±0.04)	3.77 (±0.27)
**pCCW12**[a]	0.55 (±0.03)	0.01 (±0.00)	1.00 (±0.08)	1.00 (±0.10)
**pCCW12$_{C}$**	1.31(±0.03)	0.04 (±0.00)	0.39 (±0.22)	0.63 (±0.08)
**J23117**[b]	1.00 (±0.09)	1.00 (±0.43)	0.11 (±0.01)	0.07 (±0.01)
**J23118**	2.22 (±0.19)	5.37 (±2.07)	0.08 (±0.01)	0.08 (±0.01)
**J23119**	12.33 (±1.21)	159.85 (±9.73)	0.06 (±0.01)	0.07 (±0.01)

[a] In *S. cerevisiae*, transcription and translation data are normalized to the native *CCW12* promoter

[b] In *E. coli*, transcription and translation data are normalized to the J23117 promoter

Supplementary Table S7. Relative cross-kingdom transcriptional and translational activities of hybrid promoters in *E. coli* and *S. cerevisiae*, related to Fig. 3A and 3B.

Promoter	*E. coli*		*S. cerevisiae*	
	**Relative mRNA expression level**	**Relative fluorescence intensity**	**Relative mRNA expression level**	**Relative fluorescence intensity**
**pTEF1**	0.83 (±0.34)	0.01 (±0.00)	1.44 (±0.11)	6.00 (±0.10)
**pTEF1$_C$**	0.96 (±0.01)	0.02 (±0.00)	0.34 (±0.01)	3.71 (±0.18)
**pTEF1$_C$-MTIE**	0.24 (±0.04)	0.09 (±0.01)	0.34 (±0.02)	4.45 (±0.31)
**pCCW12**[a]	0.72 (±0.17)	0.01 (±0.00)	1.00 (±0.01)	1.00 (±0.04)
**pCCW12 $_C$**	0.99 (±0.24)	0.04 (±0.00)	0.28 (±0.01)	0.65 (±0.05)
**pCCW12 $_C$-MTIE**	1.15 (±0.15)	0.25 (±0.03)	0.26 (±0.05)	1.16 (±0.05)
**pTDH3**	1.17 (±0.46)	0.02 (±0.00)	1.91 (±0.34)	7.91 (±0.31)
**pTDH3$_C$**	1.43 (±0.16)	0.09 (±0.01)	0.68 (±0.13)	2.42 (±0.19)
**pTDH3$_C$-MTIE**	1.65 (±0.07)	0.27 (±0.10)	0.56 (±0.07)	2.26 (±0.24)
**pTEF1$_C$-J23117**	0.52 (±0.20)	0.06 (±0.01)	0.21 (±0.03)	0.22 (±0.01)
**pTEF1$_C$-J23118**	2.48 (±0.45)	0.22 (±0.01)	0.27 (±0.04)	2.60 (±0.07)
**pTEF1$_C$-J23119**	3.50 (±0.13)	23.34 (±0.60)	0.33 (±0.03)	2.67 (±0.20)
**pCCW12$_C$-J23117**	1.43 (±0.13)	0.14 (±0.02)	0.22 (±0.01)	0.14 (±0.01)
**pCCW12$_C$-J23118**	3.24 (±0.93)	1.47 (±0.05)	0.23 (±0.05)	0.62 (±0.01)
**pCCW12$_C$-J23119**	4.28 (±0.70)	66.76 (±1.67)	0.36 (±0.16)	1.02 (±0.06)
**pTDH3$_C$-J23117**	1.39 (±0.34)	0.21 (±0.047)	0.36 (±0.03)	0.52 (±0.02)
**pTDH3$_C$-J23118**	3.87 (±1.13)	2.84 (±0.55)	0.42 (±0.02)	2.35 (±0.27)
**pTDH3$_C$-J23119**	6.92 (±2.07)	94.22 (±21.18)	0.45 (±0.02)	2.88 (±0.56)
**J23117**[b]	1.00 (±0.30)	1.00 (±0.13)	0.12 (±0.02)	0.07 (±0.01)
**J23118**	2.08 (±0.18)	5.37 (±2.07)	0.08 (±0.01)	0.08 (±0.01)
**J23119**	11.55 (±1.13)	159.85 (±9.73)	0.07 (±0.01)	0.07 (±0.01)

[a] In *S. cerevisiae*, transcription and translation data are normalized to the native *CCW12* promoter
[b] In *E. coli*, transcription and translation data are normalized to the J23117 promoter

Supplementary Table S8. β-Galactosidase activities of representative promoters in *E. coli* and *S. cerevisiae*, related to Fig. 3C.

Promoter	β-galactosidase activity (Miler Units)	
	E. coli	***S. cerevisiae***
**pCCW12**	0.56 (±0.08)	5.79 (±0.31)
**pTDH3**	1.19 (±0.01)	16.46 (±0.15)
**pTEF1$_C$-J23117**	6.02 (±0.57)	2.49 (±0.52)
**pTEF1$_C$-J23118**	10.65 (±0.23)	6.35 (±0.16)
**pTEF1$_C$-J23119**	15.81 (±0.53)	7.57 (±0.12)
**J23117**	4.47 (±0.31)	ND
**J23119**	24.37 (±1.64)	ND

ND, not detected.

Supplementary Table S9. PDV production correlates with hybrid promoter strength in *E. coli* and *S. cerevisiae*, related to Fig. 4C.

Strain	PDV-producing vector	PDV titer (mg/L)	Cell growth (OD_{600})
E. coli	J23117/vioABE (Weak control)	27.12 (±1.29)	5.60 (±0.32)
	pTDH3c–J23117/vioABE (Weak)	10.68 (±0.75)	5.42 (±0.26)
	pTDH3c–J23119/vioABE (Strong)	33.60 (±2.00)	5.39 (±0.37)
	J23119/vioABE (Strong control)	45.55 (±1.64)	5.76 (±0.16)
S. cerevisiae	pCCW12/vioABE (Weak control)	3.49 (±0.60)	27.07 (±0.57)
	pTDH3c–J23117/vioABE (Weak)	1.91 (±0.16)	24.47 (±0.92)
	pTDH3c–J23119/vioABE (Strong)	4.99 (±0.57)	24.13 (±0.23)
	pTDH3/vioABE (Strong control)	6.65 (±0.26)	25.04 (±0.45)

Supplementary Table S10. Relative fluorescence intensities of representative promoters in *C. glutamicum* and *P. pastoris*, related to Fig. 5.

**Promoter**	**Relative fluorescence intensity**	
	C. glutamicum	***P. pastoris***
**pH36 (Native promoter of *C. glutamicum*)**	1.00 (±0.22)	-
**pTEF1C-J23117**	0.27 (±0.02)	0.04 (±0.02)
**pTEF1C-J23118**	0.53 (±0.01)	0.14 (±0.02)
**pTEF1C-J23119**	22.67 (±1.60)	0.46 (±0.13)
**pGAP (Native promoter of *P. pastoris*)**	-	1.00 (±0.23)

Supplementary Materials and Methods 1. 5′ Rapid Amplification of cDNA Ends (5′ RACE).

To determine the TSSs of the hybrid promoters, 5′ rapid amplification of cDNA ends (5′ RACE) was performed using the SMARTer® RACE 5′/3′ Kit (TaKaRa Bio, Otsu, Japan) according to the manufacturer's instructions. *E. coli* cells were cultured in LB medium at 37 °C with shaking at 250 rpm for 24 h, and *S. cerevisiae* cells were cultured in YSC-URA medium supplemented with 2% glucose at 30 °C with shaking at 250 rpm for 24 h. Cells were harvested at an OD_{600} of 4. Total RNA was isolated using the RNeasy Mini Kit (Qiagen, Hilden, Germany). Prior to RNA purification, *E. coli* cells were subjected to enzymatic lysis, whereas *S. cerevisiae* cells were mechanically disrupted with Lysing Matrix C (MP Biomedicals, Irvine, CA, USA) using a FastPrep-24 5G homogenizer (MP Biomedicals, Irvine, CA, USA). To eliminate contaminating genomic DNA, RNA samples were treated with RNase-free DNase I (Thermo Fisher Scientific, Carlsbad, CA, USA) at 37 °C for 1 h. RNA integrity was verified by 2% agarose gel electrophoresis, and RNA purity was evaluated based on the A260/A280 ratio (>1.8). RNA concentrations were determined using a NanoDrop spectrophotometer (Thermo Fisher Scientific, Carlsbad, CA, USA).

For 5′ RACE cDNA synthesis, total RNA was reverse-transcribed using the SMARTer® RACE 5′/3′ Kit following the manufacturer's protocol. Briefly, the reaction mixture was incubated at 72 °C for 3 min and at 42 °C for 2 min, followed by reverse transcription at 42 °C for 90 min and enzyme inactivation at 70 °C for 10 min. PCR amplification was performed using the Universal Primer Mix (UPM) and the gene-specific primer mCherry-5RACE-GSP1. Touchdown PCR was carried out under the following conditions: 5 cycles of 94 °C for 30 s and 72 °C for 3 min; 5 cycles of 94 °C for 30 s, 70 °C for 30 s, and 72 °C for 3 min; and 30 cycles of 94 °C for 30 s, 68 °C for 30 s, and 72 °C for 3 min. PCR products were cloned into the vector provided with the SMARTer® RACE 5′/3′ Kit and subsequently analyzed by Sanger sequencing.

The first nucleotide located immediately downstream of the SMARTer II A Oligonucleotide sequence was designated as the TSS. Primer sequences used for 5′ RACE are listed in Table S4.

Supplementary Materials and Methods 2. RNA secondary structure prediction.

RNA secondary structures of the yeast 5′ untranslated regions (5′-UTRs) were predicted using RNA Folding Form V2.3 available through the UNAFold web server. For each promoter, the transcript sequence spanning from the experimentally determined *S. cerevisiae* TSS (mapped via 5′ RACE) to the nucleotide immediately upstream of the ATG start codon was used as the input. Structure predictions were performed using the default settings, and the predicted structure with the lowest Gibbs free energy (ΔG) was used for subsequent analysis.

References

1. Zuo, W., Yin, G., Zhang, L., Zhang, W., Xu, R., Wang, Y., Li, J. and Kang, Z. (2025) Engineering artificial cross-species promoters with different transcriptional strengths. *Synth Syst Biotechnol*, **10**, 49-57.
2. Zhang, P., Du, Q., Wang, Y., Wei, L. and Wang, X. (2025) Systematic representation and optimization enable the inverse design of cross-species regulatory sequences in bacteria. *Nat Commun*, **16**, 1763.
3. Patel, J.R., Oh, J., Wang, S., Crawford, J.M. and Isaacs, F.J. (2022) Cross-kingdom expression of synthetic genetic elements promotes discovery of metabolites in the human microbiome. *Cell*, **185**, 1487-1505 e1414.
4. Yuan, J., Mo, Q. and Fan, C. (2021) New Set of Yeast Vectors for Shuttle Expression in Escherichia coli. *ACS Omega*, **6**, 7175-7180.
5. Kushwaha, M. and Salis, H.M. (2015) A portable expression resource for engineering cross-species genetic circuits and pathways. *Nat Commun*, **6**, 7832.
6. Mumberg, D., Muller, R. and Funk, M. (1995) Yeast vectors for the controlled expression of heterologous proteins in different genetic backgrounds. *Gene*, **156**, 119-122.
7. Edwards, S.R. and Wandless, T.J. (2010) Dicistronic regulation of fluorescent proteins in the budding yeast Saccharomyces cerevisiae. *Yeast*, **27**, 229-236.
8. Choi, J. W., Yim, S. S., and Jeong, K. J. (2018). Development of a high-copy-number plasmid via adaptive laboratory evolution of Corynebacterium glutamicum. *Applied Microbiology and Biotechnology*, **102(2)**, 873-883.
9. Choi, Y., Lin, Y., and Kang, N. K. (2026). Systematic metabolic engineering of Komagataella phaffii for methanol-to-lactate bioconversion. *Bioprocess and Biosystems Engineering*, 1-10.
